# MicroRNA Expression Profiling in the Prefrontal Cortex: Putative Mechanisms for the Cognitive Effects of Adolescent High Fat Feeding

**DOI:** 10.1038/s41598-018-26631-x

**Published:** 2018-05-29

**Authors:** Marie A. Labouesse, Marcello Polesel, Elena Clementi, Flavia Müller, Enni Markkanen, Forouhar Mouttet, Annamaria Cattaneo, Juliet Richetto

**Affiliations:** 1 Physiology and Behavior Laboratory, Swiss Federal Institute of Technology (ETH) Zurich, Schwerzenbach, Switzerland; 2 Department of Psychiatry, Columbia University, New York City, USA; 3 Institute of Veterinary Pharmacology and Toxicology, University of Zurich – Vetsuisse, Zurich, Switzerland; 4 Institute of Anatomy, University of Zurich, Zurich, Switzerland; 5 Biological Psychiatry Laboratory, IRCCS Fatebenefratelli San Giovanni di Dio, Brescia, Italy; 6 Stress, Psychiatry and Immunology Laboratory, Department of Psychological Medicine, Institute of Psychiatry, King’s College London, London, UK

## Abstract

The medial prefrontal cortex (mPFC), master regulator of higher-order cognitive functions, is the only brain region that matures until late adolescence. During this period, the mPFC is sensitive to stressful events or suboptimal nutrition. For instance, high-fat diet (HFD) feeding during adolescence markedly impairs prefrontal-dependent cognition. It also provokes multiple changes at the cellular and synaptic scales within the mPFC, suggesting that major transcriptional events are elicited by HFD during this maturational period. The nature of this transcriptional reprogramming remains unknown, but may include epigenetic processes, in particular microRNAs, known to directly regulate synaptic functions. We used high–throughput screening in the adolescent mouse mPFC and identified 38 microRNAs differentially regulated by HFD, in particular mir-30e-5p. We used a luciferase assay to confirm the functional effect of mir-30e-5p on a chosen target: Ephrin-A3. Using global pathway analyses of predicted microRNA targets, we identified biological pathways putatively affected by HFD. Axon guidance was the top-1 pathway, validated by identifying gene expression changes of axon guidance molecules following HFD. Our findings delineate major microRNA transcriptional reprogramming within the mPFC induced by adolescent HFD. These results will help understanding the contribution of microRNAs in the emergence of cognitive deficits following early-life environmental events.

## Introduction

The medial prefrontal cortex (mPFC) is a complex and highly interconnected brain region critically implicated in the regulation of higher-order cognitive functions such as working memory, attention or behavioral flexibility^1^. One key feature defining this brain region is its remarkable maturational trajectory; unlike most other cortical areas, maturation in the prefrontal cortex continues until late adolescence, thus being the last brain region to achieve full maturity in humans^2–4^ and rodents^5–7^. Therefore, adolescence is a period of extensive remodeling in morphology, functional connectivity and gene expression patterns within the mPFC, and this protracted maturation is thought to confer an extended period of plasticity that supports experience-dependent learning^7,8^. At the same time, however, such plasticity is also thought to provide a basis for developmental disruption by early-life environmental insults^9^. Human and rodent studies both have shown that drug use or psychosocial stress associate with a higher risk of developing mental illness or behavioral dysfunctions when the exposure occurs during adolescence^10–13^. More recent work has shown that unhealthy nutrition during adolescence, in particular consumption of high-fat or high-sugar diets, also associates with deficits in executive functioning, and with a reduction in the volumes of frontal cortical regions in humans^14–18^. This is alarming as the quality of the human diet has deteriorated in the past few decades, now incorporating increasing levels of processed foods, artificial additives, refined sugars, and unhealthy dietary fats^19,20^, which may in turn negatively affect the mPFC. Adolescents in particular have a tendency to follow dietary guidelines less closely than adults^21–24^. Importantly, they are at a vulnerable time point in terms of nutritional training, when they begin making their own decisions about what to eat, yet are still influenced by peer-pressure and media which tend to favor less healthy nutritional options^7,23–26^.

In previous studies, our groups and others have shown that mice fed high-fat diets (HFD), or high-fat high-sugar diets, develop particularly potent prefrontal-dependent cognitive deficits when the dietary exposure begins during adolescence as compared to adulthood^27–31^. We also showed that adolescent HFD is associated with multiple and various changes at the cellular and synaptic scales within the mPFC, including modulation of α-amino-3-hydroxy-5-methyl-4-isoxazolepropionic acid (AMPA) and N-Methyl-D-aspartate (NMDA) neurotransmission, impairments in synaptic plasticity, or reductions in interneuron-specific protein levels. Notably, some of these neuronal alterations are not observed with a similar dietary exposure during adulthood. Such findings suggest that, at the molecular scale, there may be a number of transcriptional reprogramming events that occur in response to HFD in the mPFC during adolescence, which in turn would modulate expression levels of multiple neuronal proteins and affect mPFC function.

The nature of these transcriptional reprogramming events remains unknown, but could include epigenetic mechanisms that allow modifying gene activity without altering the DNA code. Indeed, accumulating evidence^32–35^ indicates that epigenetic factors represent a key mechanism linking early-life stress or suboptimal nutrition with changes in brain function^36^. For instance, early exposure to HFDs was shown to recruit such epigenetic regulatory machineries and to induce the appearance of metabolic abnormalities^37,38^; such effects could in turn also be valid for the regulation of synaptic and cognitive functions by HFD. The repertoire of epigenetic regulators is large and multi-layered, yet key candidates would include microRNAs (miRNAs), a family of small non-coding RNAs that regulate gene function by inhibiting the expression of their target mRNAs^39,40^. MiRNAs play important regulatory roles in a variety of cellular and subcellular functions^41^ and are now recognized as key modulators of dendritic and synaptic maturation and synaptic activity^42–44^, which in turn will modify cognitive performance^45,46^. Several studies have indeed characterized the contribution of prefrontal miRNAs to PFC-dependent tasks such as fear extinction and transition to alcohol addiction^46,47^. For instance, prefrontal mir-128b, whose expression dynamically changes across behavioral training in mice, was shown to regulate expression of several plasticity-related genes within the mPFC and to regulate behavioral performance on a mPFC-dependent task^46^.

It is thus highly feasible that prefrontal miRNAs could represent a significant link between early adolescent exposure to HFD and prefrontal-dependent cognitive dysfunctions, although such hypothesis has not been examined yet. We thus set out to identify global miRNA mapping within the mPFC in mice exposed to HFD since adolescence using high–throughput screening. We also used global pathway analyses of predicted miRNA targets with the aim of identifying novel biological pathways putatively affected by adolescent HFD. Finally, we validated these analyses using qPCR to confirm the emergence of miRNA-relevant gene expression changes within a top predicted biological pathway, namely *Axon Guidance*.

## Materials and Methods

### Animals

C57BL/6 N mice were used throughout the study. C57BL/6 N male and female breeding pairs obtained from Charles River (Sulzfeld, Germany) were maintained in our animal facility to generate a sufficient number of animals for the different experimental series. All animals were kept in groups (2–3 per cage) in a temperature- and humidity-controlled (21 ± 1 °C, 55 ± 5%) vivarium under a reversed light–dark cycle (lights off: 0800 to 2000h). Only male mice were included in all experiments to avoid potential confounds arising from sexual dimorphism. All procedures were approved by the Cantonal Veterinary Office of Zurich and are in agreement with the principles of laboratory animal care in the *Guide for the Care and Use of Laboratory Animals* (National Institutes of Health Publication No. 86–23, revised 1985).

### Chronic HFD and CD feeding

Experimental diets included a HFD consisting of 60% of calories from fat or a control diet (CD) composed of 10% of calories from fat (SSNIFF Diets, Germany). Diets and water were always accessible *ad libitum* throughout all experiments. Mice had access to HFD or CD starting from postnatal day (PND) 28 for 8 weeks, and body weights were measured throughout. Hence, animals were exposed to HFD or CD throughout adolescent development, covering pre-pubertal and post-pubertal stages of maturation^7^ (for more detail see Supplementary Information).

Four cohorts of mice were included in the study: experimental series 1 (Cohorts 1 and 2) aimed at assessing behavioral phenotypes of HFD and control mice, while experimental series 2 (Cohort 3) and 3 (Cohort 4) determined changes of miRNA expression, and gene expression, respectively, in naïve animals so as to avoid the possible confounding effects of repeated behavioral testing. Behavioral and molecular analyses were conducted in adulthood.

### Spatial working memory in the Y-maze

Working memory is a special short-term memory buffer used to hold relevant information temporarily active in order to guide on-going behavior^48^. The Y-maze apparatus has been extensively described previously^28^, and the test procedure is described in the Supplementary Information.

### Discrimination reversal learning in the water T-maze

The apparatus and the test procedure are described in detail in the Supplementary Information.

Briefly, during the acquisition training, the animals were required to learn to discriminate the left and right goal arms, with only one of them leading to an escape platform hidden at the far end (right arm for half of the animals; left arm for the other half). A first habituation session was then followed by 6 trials per day sessions, conducted at an intertrial interval of 5 min. Acquisition training continued until an animal had reached criterion performance of 10 correct responses across 2 consecutive days (i.e., 10 correct out of 12 trials). Upon reaching the acquisition criterion, the location of the platform was moved to the other, previously incorrect, arm to assess reversal learning. Reversal training continued until an animal reached criterion performance once again. The percentage of correct arm choices and the errors to criterion were recorded manually and calculated for each animal during acquisition and reversal training.

### RNA Purification

Animals from Cohort 2 were sacrificed after an 8-week exposure to HFD or CD. Brains were immediately extracted from the skull and placed dorsal side up on an ice-chilled plate. The medial PFC was dissected as previously established and fully described elsewhere^49^. Analyses were performed on mPFC tissue (Bregma: +2.3 to +1.3) that included both hemispheres of anterior cingulate, prelimbic, and infralimbic subregions. Brain specimens were collected in 96-well microtiter plates kept on dry ice and allowed to freeze before storage at −80 °C until further use.

Total RNA was isolated using the Qiagen miRNeasy Mini kit (Qiagen, Italy) according to the manufacturer’s instructions, and quantified by spectrophotometric analysis. An aliquot of each sample was then treated with DNase to avoid DNA contamination. The Qiagen miRNeasy Mini kit is optimized for isolating total RNA, including all RNA molecules from 18 nucleotides upwards (miRNAs).

### Genome wide microarray analyses of prefrontal miRNAs

Whole genome microRNA analysis (GEO accession number GSE105794) was performed using the Flash Tag Biotin HSR RNA Labeling kit (Affymetrix, Italy), and the miRNA 4.1 array strips (Affymetrix), according to the manufacturer’s protocol. After hybridization, each strip was washed using the Affymetrix Fluidics Station and then scanned in the Affymetrix Imaging station to obtain. CEL files that were then used for further bioinformatics analyses. Affymetrix CEL files were imported into Partek Genomics Suite version 6.6 for data visualization, statistical testing and quality control assessment. All the samples passed the quality criteria for hybridization controls, labeling controls and 3′/5′ Metrics. Background correction was conducted using Robust Multi-strip Average (RMA)^50^ and normalization was conducted using Quantiles Normalization^51^. Subsequently, a summarization step was conducted using a linear median polish algorithm^52^ to integrate probe intensities in order to compute the expression levels for each gene transcript.

Statistical analyses were performed using the Robust MultiChip Average ANOVA statistical test to assess treatment effects. Differential miRNA expression was assessed by applying a *p*-value filter (for attribute) of p < 0.05 to the ANOVA results. To investigate the effect of HFD, we performed a linear contrast between HFD vs. CD. In this comparison, a maximum filter of *p* < 0.05 and a minimum absolute fold change cut-off of 1.2 was applied. All pre-miRNAs (i.e. immature forms of miRNAs) were excluded from further analysis. This yielded 38 dysregulated miRNAs.

### Bioinformatics analyses

In order to identify the potential gene targets of single miRNAs that were observed to be differentially expressed in HFD vs. CD, two different bioinformatics databases were used jointly: TargetScanMouse (www.targetscan.org) and miRWalk, (www.umm.uni-heidelberg.de/apps/zmf/mirwalk). As the different bioinformatics tools identified different target genes for each miRNA, we performed an overlap of the results obtained by TargetScan and miRwalk for each selected miRNA, which yielded a list of predicted targets that were identified by both TargetScan and miRwalk. MiRNAs that were not annotated in these two databases were excluded from further pathway analysis, yielding a predicted target list for 9 final miRNAs. We then performed single miRNA pathway analysis (using the predicted target gene list identified above by TargetScan and miRWalk) with Ingenuity Pathway Analysis software (Ingenuity Systems, www.ingenuity.com).

In addition, top canonical pathways predicted to be affected by the combinatory of all significantly dysregulated (and annotated) miRNAs (N.B. mir-129b was excluded from this analysis, because it was not annotated in mirPath) were assessed with mirPath software (DIANA TOOLS, 2014)^53^.

### Validation of regulated miRNAs by qRT-PCR

Affymetrix results were validated by performing qRT-PCR on selected candidate miRNAs. cDNA was synthesized by using 10 ng of total RNA with TaqMan-specific RT primers (TaqMan MicroRNA Assay, Applied Biosystems) and a TaqMan miRNA reverse transcription kit (Applied Biosystems) according to the manufacturers’ instructions. Thereafter, quantitative real-time PCR was performed using predesigned assays (TaqMan MicroRNA Assays - Applied Biosystems). PCR reactions were performed as follows: 50 °C for 2 minutes, 95 °C for 10 minutes, followed by 40 cycles of 95 °C for 15 seconds and 60 °C for 1 minute. Relative target gene expression was calculated according to the 2(-Delta Delta C(T)) method, using RnU6 as internal standard. The samples and standard curves were analyzed in triplicate. miRNA fold change data obtained by qRT-PCR was then correlated against miRNA fold change obtained by microarray on a selected 9 miRNAs that included both upregulated and downregulated miRNAs.

### Luciferase assay

The reporter plasmid was constructed by inserting Ephrin A3 (EFNA3) mRNA 3′UTR (NM_010108.1) downstream of the Firefly luciferase gene in the pEZX-MT06 vector (GeneCopoeia, Rockville, USA). HEK293T or HeLa cells were maintained in Dulbecco’s modified Eagle’s medium (DMEM) containing 10% fetal bovine serum (FBS) and Pen-Strep 1x (Gibco) and plated 24 h before transfection in 96-well plates at 10e5 cells/well. Cells were transfected with 100 ng of pEZX-MT06/EFNA3-3′UTR or pEZX-MT06 and 10 pmol of mir-30e-5p (#4464066) and negative control (#4464058) miRNAs mimics (mirVana™, Life Technologies, Zug, Switzerland) per well using Lipofectamine RNAiMAX reagent (Invitrogen) according to the manufacturer’s instructions. Two wells of each specific condition were transfected to obtain a mean luciferase activity. All the experiments were performed in triplicate (3 assays for HEK293T and 3 assays for HeLa cells). Luciferase activity was measured 24 h after transfection using the Luc-Pair Duo-Luciferase Assay Kit 2.0 (GeneCopoeia) in a Luminometer (Dynex Magellan Biosciences) according to the manufacturer’s instructions. Normalization of the data included two sequential steps. (1) normalization of Firefly luciferase activity to Renilla luciferase activity (transfection control); (2) normalization against (a) the cellular effects (e.g., RNA binding proteins, endogenous miRNAs, etc.) on luciferase activity and (b) against the exogenous effects of mir-30e-5p on the luciferase coding sequences (see Supplementary Methods for more details and^54^.

### Quantitative Real-Time RT-PCR Analyses

mRNA levels of specific genes from the *Axon Guidance* pathway were quantified by SYBR Green qRT-PCR (CFX384 real-time system, Bio-Rad Laboratories) using the SsoAdvanced Universal SYBR Green supermix (Bio-Rad Laboratories), following retrotranscription with the iScript cDNA synthesis kit (Bio-Rad Laboratories), as described in the Supplementary Information.

### Statistical analyses

All data were analyzed by Student’s *t* tests or by parametric analysis of variance (ANOVA) followed by Fisher’s least significant difference (LSD) post-hoc comparisons whenever appropriate. Statistical significance was set at *p* < 0.05. All statistical analyses were performed using the statistical software StatView (version 5.0), unless otherwise specified.

In the first experimental series (cohort 1), percent time in the novel arm was analyzed using an independent Student’s *t* test (two-tailed). Percent correct trials in the acquisition phase of the T-maze, and in the reversal phase, were analyzed using a 2 × 4 (diet × days) parametric ANOVA and 2 × 6 (diet × days) parametric ANOVA, respectively.

In the second experimental series (cohort 2), fold change in miRNA levels obtained by microarray data was analyzed using an overall ANOVA within the Affymetrix software, as described in the previous paragraph. Fold change in mir-30e-5p levels obtained by qRT-PCR analyses was analyzed using a Student’s *t* test (two-tailed). Correlative analyses between mean fold change obtained by qRT-PCR and miRNA fold change obtained by microarray were performed using Pearson’s product moment correlations on 9 selected miRNAs. The luciferase gene reporter assay was analyzed using a one-sample t-test to determine whether percent change in luciferase activity due to mir-30e-5p/EFNA3-3′UTR was significantly different from zero.

In the third experimental series (cohort 3), relative gene expression of each target gene was analyzed using a repeated measures 2 × 8 (diet × genes) ANOVA followed by *a priori* post-hoc comparisons.

### Data availability

The datasets pertaining to the miRNA profiling of the prefrontal cortex are available in the GEO repository (accession number GSE105794). The remaining datasets are available from the corresponding author on request.

## Results

### Chronic HFD throughout adolescence leads to cognitive disturbances

**Figure 1:**
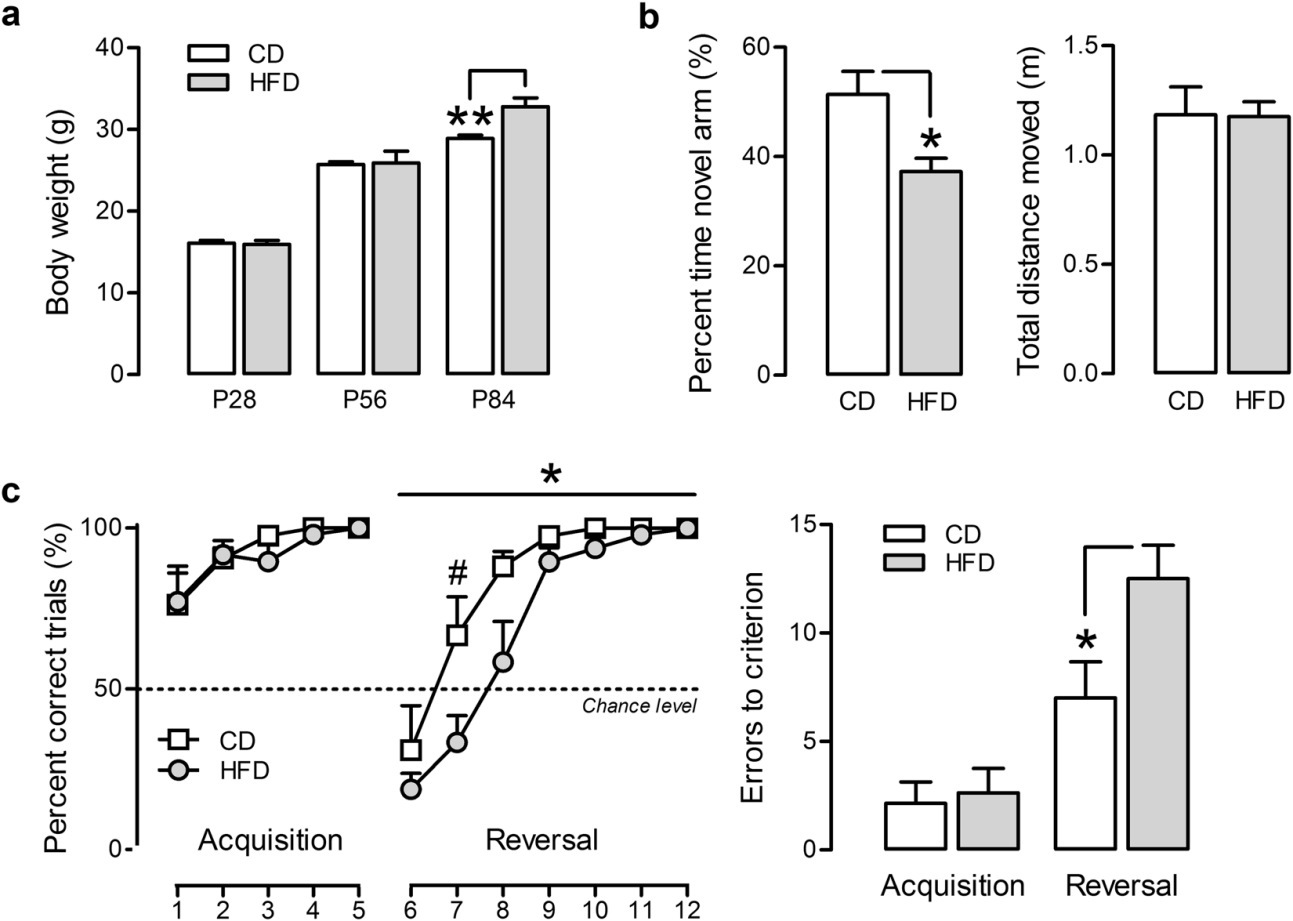
Cognitive effects of adolescent high fat diet (HFD). (**a**) Body weight of HFD and low fat diet (CD) animals at postnatal (P) day 28 (start of HFD), 56 and 84. **p < 0.01 at P84 by post-hoc comparison following significant time x diet interaction (*p < 0.01). n = 15 per diet (**b**) Left panel: Working memory indexed by the percent time spent in the novel arm during a Y-maze spatial recognition test. *p < 0.05, based on a Student**’**s t-test analysis. Right panel: Locomotor activity indexed by the total distance moved in Phase 1 of the test. n = 6–8 per diet (**c**) Cognitive flexibility assessed in a water T-maze left-right discrimination task. Left panel: Number of percent correct trials during the acquisition and reversal phase. *p < 0.05 indicating a main effect of diet in the reversal phase; ^#^*p* < 0.05 on trial 2 of the reversal phase, based on Fisher**’**s LSD post-hoc comparisons following the presence of a significant diet × trial interaction (*p* < 0.05). Right panel: Total errors to criterion in the two phases. *p < 0.05 indicating a main effect of diet in the reversal phase. n = 7–8 per diet. All data are means ± S.E.M.

As expected, chronic HFD led to an increase in body weight over time compared to control CD animals, as indicated by a significant effect of diet following 8 weeks on CD or HFD (F_(1,28)_ = 11.33, *p* < 0.01), following a significant interaction between time and diet (F_(2,56)_ = 6.77, *p* < 0.01) (Cohorts 3 and 4) (Fig. 1a). We then investigated the effects of HFD on different cognitive paradigms that depend on the integrity of the mPFC. Spatial novelty recognition memory in the Y-maze is a behavioral test that uses the natural tendency of rodents to explore novel over familiar spatial environments and that relies on (dorsal) hippocampal^55,56^ and prefrontal^57–60^ circuits. As shown in Fig. 1b, HFD throughout adolescence disrupted novel recognition as indexed by the percent time spent in the novel arm (*t*_(13)_ = 8.96, *p* < 0.05). Whilst CD control offspring exhibited a score of ∼51%, the recognition score of HFD-fed mice fell close to chance level (39%). These effects were not confounded by potential changes in locomotor activity, as total distance moved was unchanged by HFD (sample phase) (Fig. 1b).

We then tested the effects of HFD on discrimination reversal learning, a behavioral task that is known to be highly dependent on prefrontal cortex functioning^61–63^. No changes in performance were detected in the acquisition phase of the test, in which percent correct trials and total errors to criterion were similar in both treatment groups. A significant impairment in discrimination reversal learning was however detected in HFD vs. CD mice, as revealed by a main effect of diet (*F*_(1,13)_ = 5.83, *p* < 0.05) as well as a significant diet × trial interaction (*F*_(6,78)_ = 2.34, *p* < 0.05). Post-hoc comparisons confirmed that performance of HFD animals was decreased on the second trial of the reversal learning phase (F_(1,13)_ = 5.39, *p* < 0.05) with a trend on the third trial (F_(1,13)_ = 4.37, *p* = 0.057). This was further corroborated by the presence of a significant difference in the total errors to criterion between CD and HFD mice during the reversal phase of the test (t_(13)_ = 5.83, *p* < 0.05) (Fig. 1c).

### Cognitive deficits after adolescent HFD associate with changes in the expression of miRNAs in the prefrontal cortex

**Figure 2:**
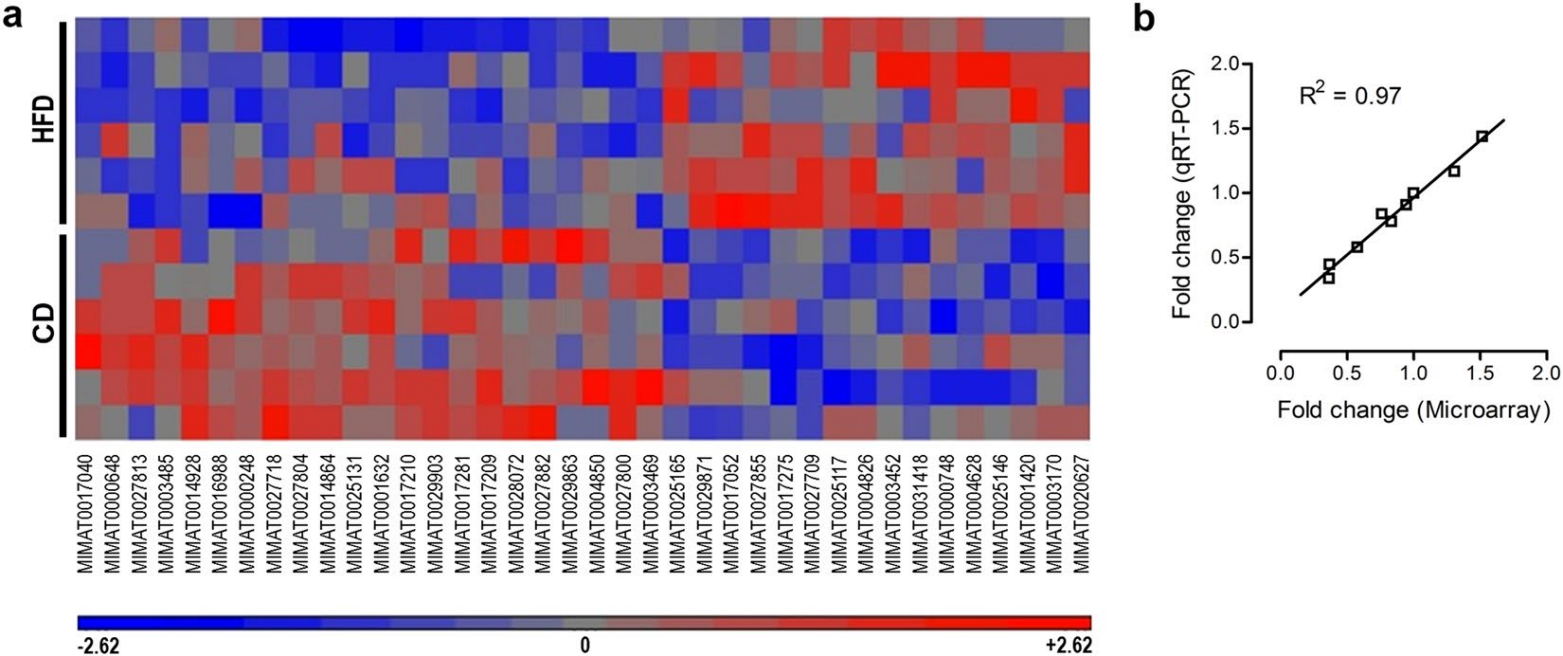
Hierarchical clustering of significantly regulated miRNAs in the PFC in adolescent high fat diet (HFD)- and low fat diet (CD)-treated mice and qRT-PCR validation. **(a)** Hierarchical clustering diagram revealing significantly regulated miRNAs using a genome wide assessment of miRNA expression by Affymetrix microarray; the analysis comprised all mature miRNAs presented in Table 1, and which were identified using a fold change cut-off value of ±1.2% and a *p*-value cut-off value of *p* < 0.05. This analysis included 38 mature miRNAs: 16 significantly upregulated and 22 downregulated miRNAs. **(b)** Pearson’s product moment correlations revealing a highly significant positive correlation between miRNA fold changes obtained by qRT-PCR and by microarray (r = 0.97, df = 7, p < 0.001) on a selected 9 miRNAs that spanned expression fold change levels from 0.3 to 1.5, thus providing statistical confirmation for the validity of microarray results. n = 6 per diet.

Next, we used genome wide assessment of miRNA expression in order to identify possible alterations in miRNA levels after chronic adolescent HFD. As listed in Table 1, we identified 38 mature miRNAs that were differentially expressed in the mPFC (fold change cutoff: ±1.2%; *p* < 0.05), including 22 significantly downregulated and 16 upregulated miRNAs. Figure 2a shows hierarchical clustering of miRNA expression changes induced by HFD.

**Table 1:** List of significantly affected miRNAs in the PFC induced by adolescent high fat diet (HFD) treatment using a genome wide assessment of miRNA expression by Affymetrix microarray.

Accession Number	Transcript ID	*p*-value	Fold-Change (HFD vs. CD)
**Significantly upregulated miRNAs - n = 16**			
MIMAT0027855	mmu-miR-6976–3p	0.0429968	1.20323
MIMAT0025165	mmu-miR-6412	0.0423496	1.20987
MIMAT0020627	mmu-miR-5119	0.0296401	1.21053
MIMAT0025117	mmu-miR-6373	0.0136909	1.21548
MIMAT0003170	mmu-miR-541-5p	0.0324367	1.21786
MIMAT0000748	mmu-miR-383-5p	0.0117387	1.22856
MIMAT0004628	mmu-miR-21a-3p	0.0281818	1.25431
MIMAT0029871	mmu-miR-7678-3p	0.0202582	1.25933
MIMAT0031418	mmu-miR-8112	0.0389069	1.26337
MIMAT0001420	mmu-miR-433-3p	0.0145485	1.27067
MIMAT0004826	mmu-miR-146b-3p	0.0128916	1.27534
MIMAT0027709	mmu-miR-6904-3p	0.0035105	1.30778
MIMAT0025146	mmu-miR-6395	0.0411032	1.32443
MIMAT0003452	mmu-miR-678	0.0369783	1.32879
MIMAT0017275	mmu-miR-467c-3p	0.0260913	1.41821
MIMAT0017052	mmu-miR-210-5p	0.0311217	1.51463
**Significantly downregulated miRNAs - n = 22 (1/2)**			
MIMAT0001632	mmu-miR-451a	0.00716281	−1.73801
MIMAT0017281	mmu-miR-511-3p	0.0232533	−1.67302
MIMAT0029863	mmu-miR-129b-3p	0.032611	−1.64229
MIMAT0014928	mmu-miR-344c-3p	0.0336578	−1.58691
MIMAT0017209	mmu-miR-541-3p	0.0216328	−1.53285
MIMAT0003469	mmu-miR-690	0.00618111	−1.52981
MIMAT0027718	mmu-miR-6909-5p	0.0147535	−1.46981
MIMAT0017040	mmu-miR-350-5p	0.0245193	−1.34234
MIMAT0014864	mmu-miR-3078-5p	0.0416764	−1.33395
**Significantly downregulated miRNAs (2/2)**			
MIMAT0003485	mmu-miR-455-5p	0.00250818	−1.31711
MIMAT0004850	mmu-miR-883b-5p	0.0340633	−1.31545
MIMAT0027804	mmu-miR-6952-5p	0.0158051	−1.30762
MIMAT0027800	mmu-miR-6950-5p	0.0299551	−1.29758
MIMAT0025131	mmu-miR-6385	0.0406697	−1.283
MIMAT0017210	mmu-miR-547-5p	0.0313436	−1.26247
MIMAT0000648	mmu-miR-10a-5p	0.0437868	−1.25814
MIMAT0016988	mmu-miR-144-5p	0.0121198	−1.25327
MIMAT0027813	mmu-miR-6956-3p	0.00668782	−1.25066
MIMAT0027882	mmu-miR-6990-5p	0.0367154	−1.24339
MIMAT0029903	mmu-miR-7687-3p	0.0348489	−1.23791
MIMAT0028072	mmu-miR-7083-5p	0.0130252	−1.22978
MIMAT0000248	mmu-miR-30e-5p	0.0303815	−1.20456

Using a fold change cutoff value of ±1.2% and a *p*-value cut-off value of *p* < 0.05, 38 mature miRNAs were identified as being differentially expressed in the PFC. n = 6 per dietary treatment.

We validated microarray results using qRT-PCR on 9 selected miRNAs that spanned expression fold change levels from 0.3 to 1.5. Pearson’s product-moment correlations revealed a highly significant positive correlation between miRNA fold changes obtained by qRT-PCR and by microarray (r = 0.97, df = 7), n = 9, p < 0.001), thus providing statistical confirmation for the validity of the microarray results (Fig. 2b).

### Adolescent HFD reduces the expression of mir-30e-5p, a miRNA with predicted gene targets involved in axon guidance and cognition

In order to identify the potential gene targets of single miRNAs we found to be differentially expressed in HFD vs CD, we performed an overlap of two different bioinformatics databases jointly: TargetScanMouse and miRWalk, that yielded a predicted target list (Supplementary Table 2) derived from the overlap of those gene lists for each miRNA that was annotated in both softwares. We then performed single miRNA Ingenuity Pathway Analysis (IPA) generating a canonical pathway analysis for each miRNA (data not shown).

**Figure 3:**
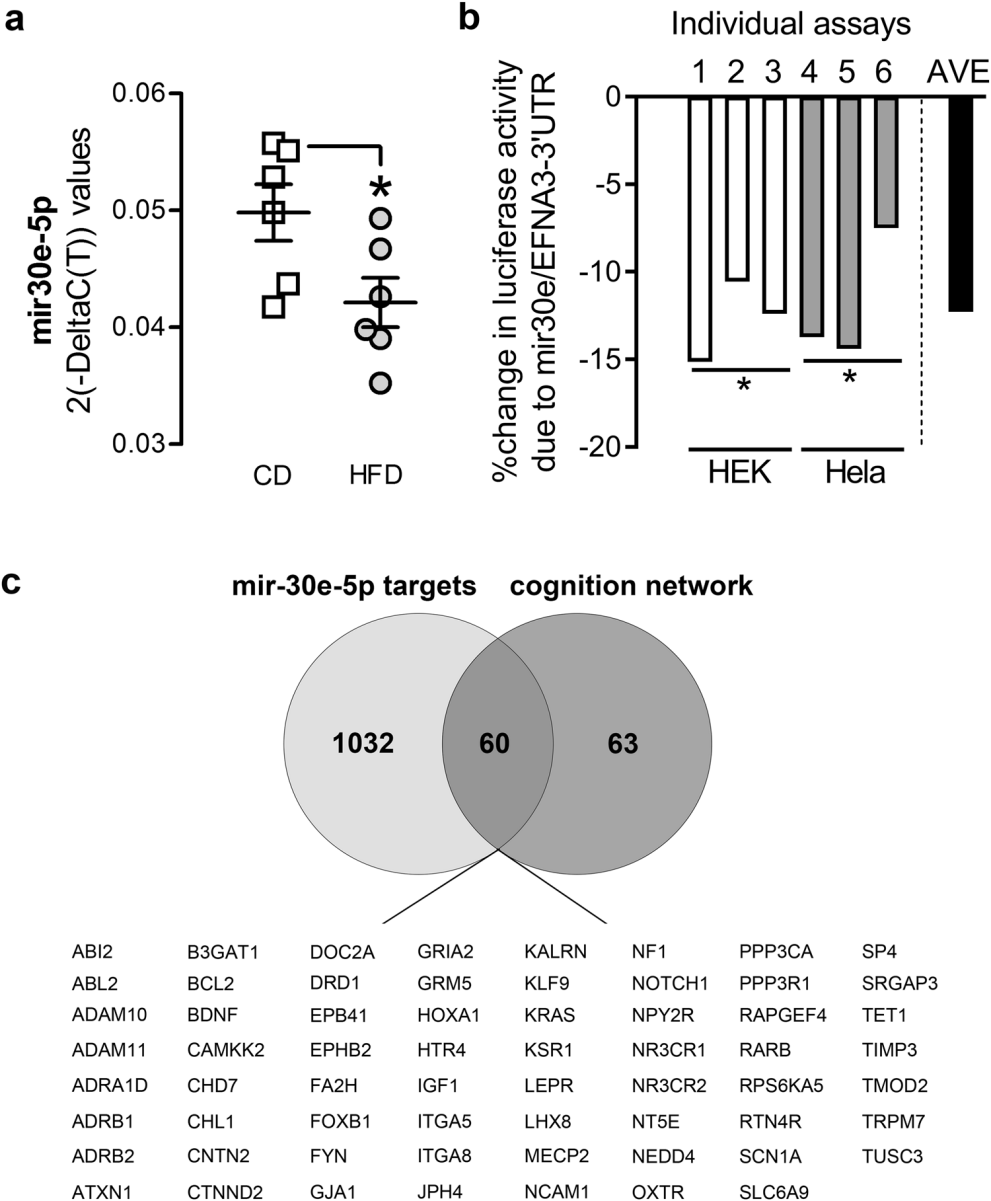
Targets and expression regulation of mir-30e-5p in the PFC induced by adolescent high fat diet (HFD) **(a)** Downregulation of mir-30e-5p expression levels, illustrated by the significantly reduced 2(-Delta(C(T)) values and revealed by a Student’s t-test analysis: *p < 0.05. n = 6 per diet. **(b)** Luciferase reporter gene assay using mir-30e-5p and EFNA3 3′UTR. Mir-30e-5p leads to a downregulation of EFNA3 (average 12% reduction in normalized luciferase activity. *P < 0.05, based on a one-sample t-test in HEK and in HeLa cells assessing difference of the mean from zero. 2 biological replicates/assay (6 assays). **(c)** Overlap analysis of mir-30e-5p molecular targets and genes belonging to the Ingenuity Pathway Analysis (IPA) ‘cognition’ network, revealing that miR-30e-5p is the first upstream regulator of the IPA ‘cognition’ network (60 genes out of 63).

Among the miRNAs that we analyzed, miR-30e-5p was particularly interesting when considering the association between HFD and cognition. Both microarray (Fig. 2a, see MIMAT0000248) and qRT-PCR (Fig. 3a, *F*_(1,10)_ = 5.75, *p* < 0.05) analyses revealed that miR-30e-5p was significantly downregulated by adolescent HFD. Among the top-5 significant canonical pathways revealed by IPA analysis, 3 were related to neuronal functions, namely *Axon guidance Signaling*, *Insulin Growth-Factor 1 (IGF 1) Signaling* and *Nerve Growth-Factor (NGF) Signaling* (Table 2). We first aimed at providing a proof-of-principle validation for the predicted targets of miR-30e-5p. We performed a luciferase gene reporter assay so as to evaluate the effects of miR-30e-5p on the expression of Ephrin A3 (EFNA3), an axon guidance molecule. We focused on EFNA3 because it was one of three axon guidance genes (top-1 canonical pathway) that was a predicted target of mir-30e-5p. The EFNA3 molecule itself also has a demonstrated role in cognition and behavior based on previous studies^64,65^ and regulation of EFNA3 expression is known to depend on microRNA-mediated mechanisms^66^. We found that mir-30e-5p led to a significant downregulation of the EFNA3 gene found in both HEK cells and HeLa cells (*t*_(2)_ = 9.55, *p* < 0.05 and *t*_(2)_ = 5.42, *p* < 0.05); Fig. 3b and Supplementary Table 3), thus in line with the predictions of the bioinformatics analyses.

**Table 2:** Ingenuity Pathway Analysis (IPA) for mir-30e-5p, a significantly downregulated miRNA in the PFC induced by adolescent high fat diet (HFD).

Name	*p*-Value	# Molecules
**Canonical Pathways**		
Axon guidance Signaling	3.88E-11	57/432
IGF-1 Signaling	1.66E-06	18/97
NGF Signaling	2.73E-05	17/107
PKCΘ Signaling in T Lymphocytes	2.82E-05	18/118
Cardiac Hypertrophy Signaling	7.11E-05	26/223
**Diseases and Disorders**		
Cancer	1.91E-24−4.60E-04	809
Gastrointestinal Disease	1.61E-17−4.59E-04	425
Organismal Injury and Abnormalities	6.23E-11−4.59E-04	419
Reproductive System Disease	6.23E-11−4.01E-04	360
Neurological Disease	3.32E-09–4.79E-04	288
**Physiological System Development and Function**		
Organismal Survival	2.77E-21–4.85E-04	278
Behavior*	2.25E-15–3.08E-04	141
Nervous System Development and Function	4.41E-15–4.75E-04	277
Tissue Development	4.41E-15–4.75E-04	361
Organismal Development	7.60E-15–4.75E-04	258
*Behavior: Top affected functions		
**SubCategories**	**Diseases or Functions Annotation**	***p*** **-Value**
Behavior	Behavior	2.25E-15
Behavior	Cognition	2.62E-11
Behavior	Learning	2.67E-10
Behavior, Nervous System Development and Function	Memory	5.40E-09

IPA analysis: (i) revealed a significant connection between the predicted targets of miR-30e-5p and three neuronal-related pathways, namely Axon guidance Signaling, IGF-1 Signaling and NGF Signaling, (ii) reported ‘behavior’ as the second top biological function affected by the targets of miR-30e-5p, (iii) indicated that targets of miR-30e-5p are implicated in cognition, learning and memory functions and (iv) revealed that miR-30e-5p is the first upstream regulator of the IPA cognition network by which 60 genes (out of 63) are predicted target molecules of miR-30e-5p (targets detailed in Fig. 3).

Furthermore, when further investigating IPA analysis for miR-30e-5p, we found that, although there is no functional evidence in the literature or experimental data regarding the correlation between miR-30e-5p and cognition, IPA reported ‘behavior’ as the second top biological function affected by the targets of miR-30e-5p (Table 2). Further detailed analysis of individual functions belonging to the behavioral network indicated that targets of miR-30e-5p are significantly implicated in cognition and learning and memory functions. Moreover, the IPA software indicated miR-30e-5p as the first upstream regulator of target-genes implicated in cognition (“IPA cognition network”), as 60 genes (out of 63) from the IPA cognition network were predicted target molecules of miR-30e-5p. Predicted targets of miR-30e-5p, which also belong to the cognition network, are reported in Fig. 3c. MiR-30e-5p thus represents a very interesting miRNA that might be implicated in some of the cognitive deficits induced by adolescent HFD.

### Global pathways analysis of all HFD-regulated miRNAs identifies biological pathways involved in brain related functions

To further identify biological events putatively affected by global miRNome changes after adolescent HFD, we used mirPath software (DIANA TOOLS) (Vlachos *et al*.^53^) on a combinatory of all significantly dysregulated miRNAs. To this purpose, only annotated miRNAs (i.e. miRNAs with known targets) were used and included 23 different miRNAs as listed in Supplementary Table 4. Such analysis allowed the identification of the top 20 canonical pathways predicted to be affected by HFD via miRNAs-related mechanisms, as shown in Table 3. Different disease-related pathways (particularly cancer-related) that are composed of a number of more specific signaling pathways were omitted from the pathway list as performed by others^67^, to avoid any bias in the interpretation of pathway analyses. Interestingly, *Axon guidance* (*p* = 1.3 E-25) yielded the highest score, with 51 genes in the pathway being potential targets of the listed miRNAs. Pathways with important roles for central nervous system (CNS) such as intracellular signaling (*mTOR Signaling*, *PI3K-Akt Signaling*, *MAPK Signaling*, *Neurotrophin Signaling*, *Wnt Signaling*), assembly and organization of nervous tissue (e.g., *Regulation of Actin Cytoskeleton*, *Focal Adhesion*, *Gap Junction*) and glutamatergic-related synaptic signaling (*Long-Term Depression*, *Glutamatergic Synapse*) resulted as the most significant. Finally, pathway analysis also included immune-related pathways (*T-cell Receptor Signaling*, *TGF-beta Signaling*, *B-cell Receptor Signaling*) and pathways related to protein processing (*Ubiquitin Mediated Proteolysis*, *Protein Processing in Endoplasmic Reticulum*) (see Table 3).

**Table 3:** DIANA Pathway Analysis of significantly regulated miRNAs in the PFC induced by adolescent high fat diet (HFD).

#	KEGG pathway	*p*-value	#genes	#miRNAs
1	Axon guidance (mmu04360)	1.29E-25	51	15
2	mTOR signaling pathway (mmu04150)	7.44E-18	27	12
3	ErbB signaling pathway (mmu04012)	6.50E-13	31	11
4	T cell receptor signaling pathway (mmu04660)	2.86E-12	34	14
5	PI3K-Akt signaling pathway (mmu04151)	2.86E-12	79	15
6	Regulation of actin cytoskeleton (mmu04810)	1.11E-11	56	15
7	MAPK signaling pathway (mmu04010)	5.98E-10	62	17
8	Ubiquitin mediated proteolysis (mmu04120)	3.65E-09	39	13
9	Neurotrophin signaling pathway (mmu04722)	4.86E-09	34	14
10	Focal adhesion (mmu04510)	4.93E-09	49	15
11	Gap junction (mmu04540)	1.17E-07	23	11
12	TGF-beta signaling pathway (mmu04350)	1.93E-07	26	9
13	Aldosterone-regulated sodium reabsorption(mmu04960)	2.49E-07	14	8
14	B cell receptor signaling pathway (mmu04662)	2.49E-07	23	13
15	Long-term depression (mmu04730)	6.26E-07	19	9
16	Glutamatergic synapse (mmu04724)	1.22E-06	30	13
17	Wnt signaling pathway (mmu04310)	2.47E-06	38	16
18	Osteoclast differentiation (mmu04380)	2.75E-06	3	12
19	Protein processing in endoplasmic reticulum(mmu04141)	3.01E-06	39	17
20	Insulin signaling pathway (mmu04910)	3.78E-06	33	11

Analysis was conducted using mirPath software (DIANA TOOLS) (Vlachos *et al*.^53^) whereby only annotated miRNAs were used. The 23 annotated miRNAs are listed in Supplementary Table 4. Such analysis allowed identifying the top 20 canonical pathways predicted to be affected by HFD via miRNA mechanisms. Different disease-related pathways (particularly cancer-related) were omitted.

### Gene expression analyses confirm downregulation of Axon guidance molecules by adolescent HFD

To provide experimental validation for our bioinformatics analyses, we then decided to evaluate whether the expression levels of genes involved in *Axon Guidance* signaling, which resulted to be the most significant pathway affected by the modulated miRNAs, were indeed affected by HFD. To do so, we performed qRT-PCR on prefrontal tissue of HFD and CD animals on selected genes involved in this pathway.

**Figure 4:**
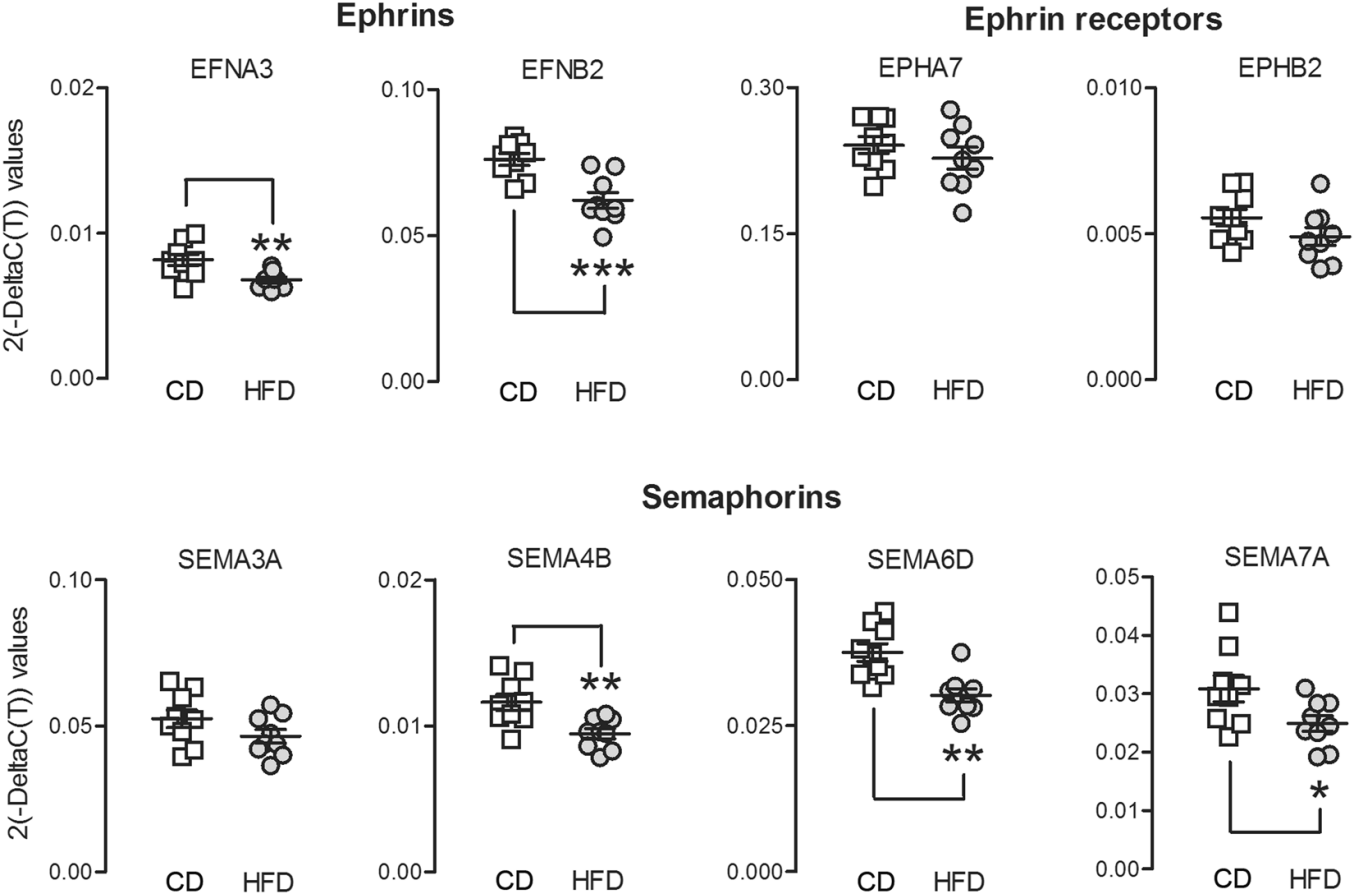
Gene expression changes of axon guidance molecules induced by high fat diet (HFD) (**a**) Gene expression levels of Ephrins A3 and B2 indexed by fold change in HFD vs. low fat diet (CD) treatments. **p < 0.01 and ***p < 0.001 by a priori post-hoc comparison (**b**) Gene expression levels of Ephrin receptors A7 and B2 (**c**) Gene expression levels of Semaphorins 3A, 4B, 6D and 7A. **p < 0.01 by a priori post-hoc comparison. n = 9 per diet. All data are means ± S.E.M.

As outlined in Supplementary Table 5, 15 miRNAs altered by HFD had one or several mRNA targets within the *Axon guidance* pathway, totaling up to 51 genes within this pathway. To this end, we chose to analyze *Axon guidance* target genes predicted by our miRNA target analyses (DIANA tools) (represented in Supplementary Figure 1). We focused our analyses on Semaphorins and Ephrins (including Ephrin receptors), as these *Axon guidance* families were both strong targets predicted by the pathway analyses. A repeated-measures ANOVA over all target genes first confirmed a global impact of HFD on *Axon guidance* gene expression, illustrated by a significant main effect of diet (*F*_(1,16)_ = 16.56, *p* < 0.001) but no significant diet × gene interaction. Using *a* *priori* post-hoc analyses to identify which genes were specifically affected, we found that Ephrin A3 (*F*_(1,16)_ = 10.05, *p* < 0.01), Ephrin B2 (*F*_(1,16)_ = 16.98, *p* < 0.001), Semaphorin 4B (*F*_(1,16)_ = 11.03, *p* < 0.01), Semaphorin 6D (*F*_(1,16)_ = 15.35, *p* < 0.01) and Semaphorin 7A (*F*_(1,16)_ = 5.26, *p* < 0.05) were significantly downregulated by HFD as compared to controls by an average 15%, while Ephrin Receptor A7, Ephrin Receptor B2 and Semaphorin 3A remained unaffected by the manipulation (Fig. 4). These analyses thus provide a proof of concept to suggest that miRNAs significantly affected by HFD may have a functional impact on gene expression, through a downregulation of some, but not all, genes within the *Axon guidance* pathway.

## Discussion

Despite the well-established effects of HFD on higher-order cognition^68^, studies had thus far essentially focused on the hippocampus, whereas the effects on the mPFC remained less well understood. Recent work, however, has shown that HFD can indeed affect various forms of cognitive functions that depend on the mPFC^27–31^. In particular, these studies have shown remarkable effects when HFD begins during adolescence, thus emphasizing that the maturing mPFC is particularly sensitive to such dietary treatments. Interestingly, these investigations have shown that HFD can affect a variety of synaptic and cellular functions within the mPFC, including interneuron function and glutamatergic neurotransmission and plasticity; yet changes at the molecular level within the mPFC remain thus far poorly characterized. In particular, given the functional changes induced by HFD within the mPFC, it would be important to identify potential regulatory mechanisms underlying such changes at the transcriptional level.

The present study first confirmed that chronic HFD consumption throughout adolescent development in mice leads to marked abnormalities in a number of cognitive tasks that are partly dependent on prefrontal functioning such as working memory and discrimination reversal learning. Our study then aimed at assessing potential changes within the prefrontal miRNome induced by HFD feeding, as miRNAs represent important transcriptional regulators^39,40^, which could potentially link HFD exposure, mPFC gene expression and, eventually, cognitive dysfunctions. In this study, we provide for the first time a global miRNA profiling of the mPFC induced by HFD feeding, showing that HFD leads to an overall remodeling of the prefrontal miRNome. Our study thus substantially extends previous reports by identifying a potential novel mechanism involved in the development of HFD-induced cognitive dysfunctions. MiRNAs are small 22-nucleotide non-coding RNAs that mediate post-transcriptional silencing of gene expression in a sequence specific manner. The capacity of single miRNAs to target different mRNAs makes them essential regulators of a variety of cellular functions^41^. Importantly, miRNAs represent critical mediators of a number of synaptic events such as dendritic subcellular localization, arborization and synapse formation and maturation^42–44^ that in turn are essential for information processing during cognitive tasks^45,46^. MiRNAs have also been identified as key epigenetic molecules that can translate environmental disturbances into lasting changes in cellular expression levels, as has been shown in a number of studies involving adolescent exposure to alcohol or cannabis^69,70^. Our results are thus in line with the current understanding that miRNAs could represent important cellular nodes that modulate gene expression following early life events to generate anomalies in cognitive functioning.

Although we did not investigate the cognitive implication of miRNA changes after HFD, we identify a number of miRNAs as potential targets for future studies. Of the 38 significantly affected miRNAs (Table 1 and Fig. 2a), mir30e-5p, mir-433-3p and mir-690 are particularly interesting because they are predicted regulators of three biological pathways with essential roles for proper neural functioning, namely *Axon guidance*, *Ephrin Receptor Signaling* and *Neurotrophin Signaling*. In particular, given its strong (predicted) capacity to regulate the expression of numerous genes implicated in cognition (see Fig. 3**)**, mir-30e-5p emerges as one of the most interesting candidate regulators of gene expression and cognitive function after HFD exposure. Interestingly, mir-30e-5p was shown to be similarly down-regulated in an animal model of temporal lobe epilepsy^71^, whereas mir-30e-5p precursor variants were shown to associate with schizophrenia^72,73^, supporting the relevance of this miRNA for neurological and neuropsychiatric disease. Examining the functional role of mir-30e-5p in the emergence of HFD-induced cognitive abnormalities would thus represent an interesting extension of the present study and a potential promising avenue for the understanding of cognitive deficits in diseases with prefrontal anomalies.

Furthermore, our study delineates molecular pathways putatively affected by miRNA events, some of which are critically involved in cognitive function. Notably, our results indicate that *Axon guidance* is the top-1 predicted pathway targeted by miRNAs after HFD. Importantly, we also identified multiple gene expression changes within *Axon guidance* molecules, in particular ephrins and semaphorins, thus providing proof-of-concept data suggesting that individual miRNA molecules affected by HFD may have functional effects on gene expression. We also provided a direct validation that mir-30e-5p (mentioned above) downregulates one of its predicted targets within the axon guidance pathway: Ephrin A3. Of note when looking at gene expression changes in the mouse mPFC after HFD, we find that although mir-30e-5p expression is reduced (Fig. 3a), EFNA3 expression is also reduced (Fig. 4), even though EFNA3 expression was downregulated by mir-30e-5p in a luciferase cell culture assay. However this seeming contradiction can easily be explained by the fact that multiple miRNAs can regulate the expression of one given mRNA molecule^74^. Also, it is important to remember that other mechanisms are likely to be involved in gene expression regulation in the *in-vivo* setting including DNA methylation and histone modifications^75,76^.

*Axon guidance* is a tightly regulated process that allows the correct pathfinding of axons during development. The majority of *Axon guidance* events converge during embryonic and early neonatal life^77^. Nevertheless, *Axon guidance* molecules such as ephrins, semaphorins, slits, netrins and a number of extracellular-matrix (ECM) proteins are also found in the adult brain, where they seem to play essential roles in synaptic plasticity, glutamatergic signaling, neuroadaptation^78–80^ or learning and memory functions^81,82^. Recent work has also shown that axon guidance molecules contribute to brain maturation during adolescence, in particular within the mPFC^5,83,84^. Importantly, emerging evidence suggests that axon guidance molecules may also be implicated in the reorganization of the brain after environmental disturbances such as exposure to drugs of abuse, brain injury^83,85–87^, and now HFD. Interestingly, *Axon guidance* was selected as one of the top canonical pathways in similar miRNA microarray studies involving animal models of temporal-lobe epilepsy^67^ or Alzheimer’s disease^88^. Our study thus extends such reports, indicating that *Axon guidance* might also play a role in the regulation of prefrontal function by HFD treatments through the involvement of miRNA mechanisms. Future studies are certainly warranted to address such possibilities.

The second top canonical pathway identified by our pathway analyses is the mTOR signaling pathway, a biological pathway involved in the coupling of nutrients and hormones with the regulation of energy-demanding cellular functions^89^. Interestingly, HFD has been shown previously to modulate hippocampal and cortical mTOR signaling^90,91^. In the brain, the mTOR pathway is known to play a role in the regulation of synaptic plasticity and cognitive functions^89,92^. Based on our findings, future studies are therefore warranted to determine a possible link between HFD, miRNAs, and mTOR signaling.

Moreover, our analyses implicate miRNAs in modulating canonical pathways with known roles in neural and cognitive functions. Notably, two related glutamatergic pathways, namely *Long-term Depression* (LTD) and *Glutamatergic Synapse*, emerged from such analyses, consistent with previous studies identifying defects in glutamatergic neurotransmission and plasticity in the mPFC of HFD mice^28^.

Notably, the present results corroborate previous research delineating the effects of HFD on the miRNome of peripheral^93^ or hypothalamic^94^ tissues. We identify a number of similarities in the miRNA profiles of hypothalamic and prefrontal tissue after HFD. For example, mir-30e was significantly affected by HFD in both brain regions. Moreover, biological pathways implicated in neurodevelopment such as *Axon Guidance* and *Ephrin Receptor Signaling*, as well as the *PI3K/Akt* and *mTOR signaling* pathways were commonly recognized as some of the top canonical events putatively affected by miRNAs in these models, suggesting that these cellular pathways might be particularly sensitive to miRNA-dependent signaling in CNS tissue after HFD.

Our present findings are also in line with other studies showing the impact of negative environmental events during adolescence on the prefrontal miRNome. In a study in mice exposed to cocaine during adolescence^87^, the authors identified similar changes in miRNAs regulating the *Axon Guidance* and *Wnt signaling* pathways^95^. Interestingly, both pathways are known to be involved in the development and maturation of the mPFC. Although speculative, all these studies together suggest that miRNAs, by affecting brain maturational processes, could represent critical contributors to the emergence of behavioral and cognitive deficits following adolescent exposure to environmental disruptors.

Admittedly, our study does not establish a direct causal link between the observed changes in the expression levels of miRNAs and cognitive abnormalities. Nonetheless, the use of available bioinformatic tools provided valuable insights and allowed us to speculate on potential biological networks possibly affected by miRNAs. Also, our luciferase gene reporter assay allowed to provide a proof-of-concept that at least some of the predictions of our bioinformatics analyses are valid; i.e. we showed that EFNA3 is a target for miR-30e-5p. However, more targeted approaches to examine the functional contribution of individual miRNAs are clearly necessary to fully demonstrate the relevance of distinct miRNAs in the emergence of the cognitive deficits described herein. In addition, although beyond the scope of this study, it would be interesting to determine whether changes in miRNA expression and miRNA-regulated gene expression induced by HFD would (partly or fully) recover following a return to a regular diet, as recently suggested by others^96^.

The present study will be relevant to the clinical appreciation and treatment of cognitive dysfunctions in obesity and in populations that exhibit poor dietary habits, particularly given the dramatic rise in such phenomenon in the past decades^97^. Notably, our results may have broader implications that reach beyond one’s individual experience of feeding. Indeed, a number of recent studies have also revealed that the metabolic effects of HFD can be transmitted across generations^98^. Given that epigenetic transmission of behavioral traits are known to partly depend on mechanisms involving germ-line non-coding RNAs such as miRNAs^99^, the cognitive and miRNA effects of HFD described herein might potentially be transmitted to subsequent generations, although such hypothesis requires investigation.

In conclusion, our results provide the first experimental evidence for the existence of dysregulated prefrontal miRNA expression after HFD exposure and identify target biological pathways putatively affected by miRNAs that include essential neural functions such as *Axon guidance*. Although further functional studies are required, our findings draw attention to the potential involvement of miRNAs in the development of cognitive deficits, and their implications for obesity and for brain disorders with prefrontal components.

## Electronic supplementary material

Supplementary Information
