# Supplementary material for: MicroRNA Expression Profiling in the Prefrontal Cortex: Putative Mechanisms for the Cognitive Effects of Adolescent High Fat Feeding
Source: Sci Rep. 2018 May 29;8:8344. doi: 10.1038/s41598-018-26631-x (PMC5974184; doi:10.1038/s41598-018-26631-x)
Supplement: Supplementary file 1 — Supplementary Information [file 41598_2018_26631_MOESM1_ESM.doc]

**Supplementary Information**

**MicroRNA Expression Profiling in the Prefrontal Cortex: Putative Mechanisms for the Cognitive Effects of Adolescent High Fat Feeding**

Marie A. Labouesse^1,2^, Marcello Polesel^4^, Elena Clementi^3^, Flavia Müller^3^, Enni Markkanen^3^, Forouhar Mouttet^1^, Annamaria Cattaneo^5,6^, Juliet Richetto^3^,*

^1^Physiology and Behavior Laboratory, Swiss Federal Institute of Technology (ETH) Zurich, Schwerzenbach, Switzerland.

^2^Department of Psychiatry, Columbia University, New York City, USA

^3^Institute for Veterinary Pharmacology and Toxicology, University of Zurich – Vetsuisse

^4^Institute of Anatomy, University of Zurich, Zurich, Switzerland

^5^Biological Psychiatry Laboratory, IRCCS Fatebenefratelli San Giovanni di Dio, Brescia, Italy.

^6^Stress, Psychiatry and Immunology Laboratory, Department of Psychological Medicine, Institute of Psychiatry, King's College London, London, UK.

Supplementary Materials and methods

***Chronic HFD and CD feeding***

Mice had access to HFD or CD starting from postnatal day (PND) 28 for 8 weeks, and body weights were measured throughout. Hence, animals were exposed to HFD or CD throughout adolescent development, covering pre-pubertal and post-pubertal stages of maturation ^1^. These stages are defined based on the gradual attainment of sexual maturity and age-specific behavioral discontinuities from younger to older animals ^1^. The pubescent time period (starting from PND 28) was chosen based on previous studies in mice showing that this maturational time window is highly sensitive to the deleterious effects of HFD on brain and behavioral functions ^2-4^. Furthermore, this pubescent period is characterized by a number of major maturational changes in the prefrontal cortex ^5,6^ such as synaptic pruning, myelination and axonal sprouting.

***Spatial working memory in the Y-maze***

Working memory is a special short-term memory buffer used to hold relevant information temporarily active in order to guide on-going behavior ^7^. The Y-maze was made of transparent Plexiglas and consisted of three identical arms radiating from a central triangle and spaced 120° from each other. Access to each arm from the central area could be blocked by a removable opaque barrier wall. The allocation of arms (start, familiar and novel arm) to a specific spatial location was counterbalanced across the experimental conditions. The floor of the maze was covered with sawdust bedding, which was changed between both the sample and testing phases (see below). The maze was positioned in a well-lit room enriched with distal spatial cues. A digital camera was mounted above the Y-maze apparatus. Images were captured at a rate of 5 Hz and transmitted to a PC running the EthoVision tracking software (Noldus Technology, The Netherlands). The spatial novelty recognition test in the Y-maze consisted of two phases:

- Sample phase: One of the three arms (“novel arm”) was blocked, whereas the other two (“start arm” and “familiar arm”) were freely accessible. To begin a trial, the animal was introduced at the end of the start arm and was allowed to freely explore both start and familiar arms for 5 min. Test timing was initiated once the animal entered the central area, as detected by the EthoVision tracking software. After 5 min the animal was removed and placed in a holding cage. The barrier door was removed and the sawdust flooring changed to avoid olfactory cues.
- Choice phase: The animal was reintroduced to the start arm following a retention interval of 1 min. The animal was allowed to freely explore all three arms for 5 min. The sawdust flooring was then changed in preparation for the next trial.

On each trial, the relative time spent in the novel arm during the choice phase was calculated by the formula [time spent in the novel arm]/[time spent in all arms] × 100 and used as the index for spatial novelty recognition. In addition, total distance moved in the entire maze was recorded and analyzed in order to assess general locomotor activity.

Discrimination reversal learning in the water T-maze

The apparatus consisted of a white circular tank (1 m in diameter). Opaque Plexiglas partitions were positioned vertically into the water to form three interconnected corridors in the shape of a “T” joining at the center of the water tank. A piece of cylindrical clear Plexiglas, 7 cm in diameter and 18.5 cm high, was positioned at the end of either the left or right goal arm and served as the escape platform.

In acquisition training, the animals were required to learn to discriminate the left and right goal arms, with only one of them leading to an escape platform hidden at the far end (right arm for half of the animals; left arm for the other half). Animals with strong innate preference for a particular arm (evaluated on the first habituation session, see below) were assigned that arm as the acquisition arm to prevent excessively easy reversal. In a first habituation session, animals were allowed 1) to explore the water T-maze for a few seconds and habituate to swimming in water and 2) to locating a platform (located in a different arm than any of the test arms). There were then 6 trials per daily session, conducted at an intertrial interval of 5 min. To begin a trial, the animals were placed at the beginning of the start arm and allowed up to 1 min to choose between the two arms. Once its entire body had entered into an arm, a guillotine door was lowered to prevent the animal from retracting. If the animal chose the correct arm, it was allowed to remain on the platform for 5 s, after which it was removed from the maze and returned to a holding cage. When the incorrect arm was chosen, the animal was confined to the arm for 60 s and then removed from the maze to a holding cage. Acquisition training continued until an animal had reached criterion performance of 10 correct responses across 2 consecutive days (i.e., 10 correct out of 12 trials). Upon reaching the acquisition criterion, the location of the platform was moved to the other, previously incorrect, arm to assess reversal learning. Reversal training continued until an animal reached criterion performance once again. The percentage of correct arm choices and the errors to criterion were recorded manually and calculated for each animal during acquisition and reversal training.

***Quantitative Real-Time RT-PCR Analyses***

Animals from Cohort 3 were sacrificed after an 8-week exposure to HFD or CD and brain processed as described above. mRNA levels of specific genes from the *Axon Guidance* pathway were quantified by SYBR Green qRT-PCR (CFX384 real-time system, Bio-Rad Laboratories) using the SsoAdvanced Universal SYBR Green supermix (Bio-Rad Laboratories), following retrotranscription with the iScript cDNA synthesis kit (Bio-Rad Laboratories). Samples were run in 384-well formats in triplicates, and 36B4 was used as an internal standard as its expression was not affected by the adolescent HFD manipulations.

Thermal cycling was initiated with an incubation at 95°C for 30s (polymerase activation and cDNA denaturation). After this initial step, 39 cycles of PCR were performed. Each PCR cycle consisted of heating the samples at 95°C for 15 s to enable the melting process and then for 30 s at 60°C for the annealing and extension reaction. Relative target gene expression was calculated according to the 2(-Delta Delta C(T)) method. Probe and primer sequences are summarized in **Supplementary Table 1** and were purchased from Microsynth (Switzerland).

***Luciferase assay, data analysis:***

Results from the luciferase assay were analyzed using a stringent analysis method described in Campos-Melo et al. 2014^8^ which ensures that confounding factors classically arising in luciferase assays are controlled for. In this method we first normalize the Firefly luciferase activity with the Renilla luciferase activity (transfection control). We then normalize the Relative luciferase activity using 4 different conditions:

(1) a construct containing the luciferase genes and the 3’UTR for EFNA3 + a mir-30e-5p mimic. This accounts for the cellular effects (e.g., RNA binding proteins, endogenous miRNAs, etc.) AND the exogenous effects of mir-30e-5p on the expression of luciferase coding sequences linked to the EFNA3 mRNA 3'UTR sequence.

(2) a construct containing the luciferase genes and the 3’UTR for EFNA3 + a negative miRNA mimic. This accounts for the cellular effects on the expression of luciferase coding sequences linked to the EFNA3 mRNA 3'UTR sequence.

(3) a construct containing the luciferase genes only + a mir-30e-5p mimic. This accounts for the cellular effects AND the exogenous effects of mir-30e-5p on the expression of luciferase coding sequences without the EFNA3 3'UTR sequence.

(4) a construct containing the luciferase genes only + a negative miRNA mimic. This accounts for the cellular effects on the expression of luciferase coding sequences without the EFNA3 3'UTR sequence.

A first step of normalization A=(1)/(2) allows to calculate the exogenous effects of mir-30e-5p on the expression of luciferase coding sequences linked to the EFNA3 mRNA 3'UTR sequence. In the same fashion, B=(3)/(4) represents the exogenous effects of mir-30e-5p on the expression of luciferase coding sequences without the EFNA3 3'UTR sequence.

A second step of normalization=A/B eliminates the effects on the luciferase coding sequences and singles out the sole effects of mir-30e-5p on the EFNA3 mRNA 3'UTR.

Values of relative variation in luciferase activity over 1 are considered up-regulation and values under 1 down-regulation. For ease of presentation, values are presented as percent change (values over 0 depicting upregulation and values under 0 depicting down-regulation. One sample‘s t-test was used to determine whether the mean percent change in normalized luciferase activity due to mir-30e-5p was significantly different from zero.

| **Gene** | **Forward Primer** | **Reverse Primer** |
| --- | --- | --- |
| EFNA3 | 5’-CCGAGAAGTTCCAGCGTTAC-3’ | 5’-TCCAACACGTTGATCTTCACA-3’ |
| EFNB2 | 5’-AGGAATCACGGTCCAACAAG-3’ | 5’-GTCTCCTGCGGTACTTGAGC-3’ |
| EPHA7 | 5’-GTCACTGCTGCCGGTTATG-3’ | 5’-ATGATGAAGCCGAACACCAT-3’ |
| EPHB2 | 5’-AGTTCGGCCAGATTGTCAAC-3’ | 5’-GCCTCTAGCCACTCATCCAC-3’ |
| SEMA3A | 5’-GAATGCAGTCCGAAGTCACA-3’ | 5’-CATGAATCCGTGTTCCACAG-3’ |
| SEMA7A | 5’-GACCGCTGCGTGTCTATCTA-3’ | 5’-GGCAGGTCAGGTAGTATCG-3’ |
| SEMA4B | 5’-TAGCCTCTACCAGCCTGATCTG-3’ | 5’-TGTTTGGTTGGATCTGGACTTG-3’ |
| SEMA6D | 5’-AGGTTGACAGCCATCGAAGT-3’ | 5’-GGACCACGCAGCTAGAGAAG-3’ |
| 36B4 | 5’-GCCGTGATGCCCAGGGAAGA-3’ | 5’-CATCTGCTTGGAGCCCACGTT-3’ |

**Supplementary Table 1.** List of primers used in the qRT-PCR analyses of axon guidance molecules (Figure 4)

| **mir10a-5p:** |
| --- |
| 1110067D22Rik, Abcg1, Actg1, Ank3, Ankfy1, Ankrd12, Ap4e1, Arih2, Arnt, Arrdc3, Arsj, Baz1b, Baz2b, Bbx, Bcl2l11, Bcl6, Bcr, Bdnf, Blzf1, Bmp2k, Braf, Bri3bp, Btrc, Cadm2, Calcr, Camk2b, Camk2g, Cask, Cbx5, Cdc6, Cept1, Chd6, Chl1, Cnnm4, Creb1, Crk, Csmd1, Ctnnbip1, Dazap1, Dock11, Dusp3, E130309D14Rik, E2f3, E2f7, Ebf2, Eif3j, Elavl2, Elovl6, Epha5, Epha8, Esrrg, Fbf1, Fbxo30, Fign, Flt1, Fosl2, Gabrb2, Galnt1, Gata6, Gm1568, Gtf2h1, H3f3b, Has3, Homez, Hoxa1, Hoxa3, Hoxb3, Hoxd10, Hspa12a, Igsf1, Inhbb, Ino80d, Irs4, Jarid2, Kcna6, Kctd17, Kdsr, Kl, Klc2, Klf11, Kpnb1, Lancl1, Lhfpl4, Lrig2, Ltbp1, Map3k2, Map3k7, Mapkbp1, Mapre1, Mbnl3, Mtf1, Mtf2, Mycbp, Nav1, Ncoa6, Ncor2, Nfasc, Nfat5, Nfatc2, Npas3, Nr2c2, Nr5a2, Nr6a1, Nufip2, Nup50, Odz2, Patl1, Pde4a, Pea15a, Pias1, Ppara, Prtg, Pten, Purg, Rap2a, Rc3h2, Rgs8, Rhpn2, Rorb, Rprd1a, Rqcd1, Sdc1, Serf2, Sertad4, Sh3d19, Shank3, Six4, Slc25a1, Slc41a2, Smap1, Smcr7l, Smurf1, Snx4, Sobp, Spag9, Spty2d1, St6galnac6, Styk1, Syne1, Tbx5, Tcfap2c, Tiam1, Tmem183a, Tmem20, Tmod1, Tnrc6b, Trim39, Trim66, Usf2, Usp12, Usp46, Wdr26, Wdr48, Wnt9b, Zdhhc21, Zfp367, Zfp608, Zmynd11, Zxdc |
| **mir30e-5p:** |
| 0610040J01Rik, 1110003E01Rik, 1110032A04Rik, 1700047I17Rik2, 2010011I20Rik, 2010109K11Rik, 2210012G02Rik, 2310014H01Rik, 2310028O11Rik, 2310046O06Rik, 2410042D21Rik, 2610109H07Rik, 2810046L04Rik, 3110043O21Rik, 4921524J17Rik, 4921528I01Rik, 4931406P16Rik, 4931408A02Rik, 4933426M11Rik, 4933433P14Rik, 5031414D18Rik, 5430411K18Rik, 8430427H17Rik, 9330159F19Rik, 9930021J03Rik, A230046K03Rik, Abat, Abcb7, Abcc4, Abcd2, Abi2, Abl1, Abl2, Acap2, Acp2, Actc1, Actn1, Actr1a, Acvr1, Adam10, Adam11, Adam12, Adam19, Adam22, Adam9, Adamts3, Adamts6, Adamts9, Ado, Adra1d, Adra2a, Adra2b, Adrb1, Adrb2, Afap1l2, Aff4, Ahnak, Akap11, Alpk3, Amotl2, Ankhd1, Ankrd17, Ano4, Ap2a1, Ap3m1, Ap3s1, Ap4e1, Apba1, Arhgef3, Arhgef6, Arid3a, Arid4a, Arid4b, Arid5b, Arih1, Arl15, Arl4c, Arl6ip6, Asah1, Asb3, Asb4, Ascc3, Asxl3, Atad2, Atad2b, Atf1, Atf7ip2, Atg5, Atl2, Atl3, Atp2a2, Atp2b1, Atp2b2, Atp6v0d1, Atp6v1c1, Atp8a1, Atp8b1, Atp8b2, Atxn1, Avl9, AW555464, Azin1, B230120H23Rik, B230208H17Rik, B3galt6, B3gat1, B3gnt5, B4galt5, B4galt6, B630005N14Rik, Bach1, Bach2, Bahd1, Baz1a, Baz2b, Bbx, BC021891, BC031353, BC037034, Bcl11a, Bcl11b, Bcl2, Bcl2l11, Bcl2l13, Bcl6, Bcor, Bdnf, Bdp1, Bean1, Becn1, Bend3, Bnc1, Bnc2, Bnip3l, Brap, Brd1, Brwd1, Brwd3, Bsn, Btaf1, Btbd10, C230081A13Rik, C530008M17Rik, C77080, Cacna1d, Cadps, Calb2, Calcr, Calu, Camk2d, Camk2n1, Camkk2, Camsap1, Camta1, Cand1, Cant1, Capn5, Capza1, Cars, Cbfb, Cblb, Cbx2, Ccdc117, Ccdc120, Ccdc43, Ccdc6, Ccdc97, Ccne2, Ccnf, Ccnjl, Ccnk, Ccnt2, Cdc37l1, Cdca7, Cdh20, Cdk12, Ceacam1, Ceacam2, Cecr2, Cecr6, Celf3, Celf4, Celf5, Celsr3, Cep170, Cep350, Cer1, Cfl2, Chd1, Chd5, Chd7, Chd9, Chfr, Chic1, Chka, Chl1, Chst1, Chst12, Chst2, Clec14a, Clock, Clrn1, Cmpk2, Cntn2, Cntn4, Cog3, Col13a1, Col9a3, Cops7b, Coq3, Cotl1, Cpeb2, Cpeb3, Cpne5, Cpne8, Cpsf6, Crk, Cry2, Csnk1a1, Csnk1g1, Ctdspl2, Cth, Cthrc1, Ctnnd2, Cul2, Cyb561, Cyb5b, Cyp24a1, Cysltr1, D0H4S114, D15Ertd621e, D19Wsu162e, D3Bwg0562e, D630045J12Rik, Dab1, Dach2, Dact1, Dagla, Dcbld1, Dcp1a, Dcp2, Dcun1d1, Dcun1d3, Dcx, Ddah1, Ddit4, Ddx19b, Ddx46, Dennd2c, Dennd5b, Dexi, Dgkq, Dgkz, Dhx40, Dio2, Dip2b, Dlg2, Dlg5, Dlgap4, Dll4, Dmd, Dnajc13, Dnmt3a, Doc2a, Dock7, Dolpp1, Dpy19l1, Dpysl2, Drd1a, Dsc2, Dstyk, Dtna, E2f3, E2f7, Eaf1, Ebf3, Eda, Edc3, Edem3, Ednra, Ednrb, Eea1, Eed, Efna3, Efr3a, Eif2c1, Eif2c2, Eif5a2, Elavl3, Elavl4, Elfn2, Ell, Ell2, Elmo1, Elmod2, Elovl5, Eml1, Eml4, Epb4.1, Epb4.1l3, Epb4.1l4b, Epb4.1l5, Epc1, Epc2, Ephb2, Erc2, Erlin1, Ermap, Esco1, Esrrg, Etaa1, Extl2, Eya2, Fa2h, Faf2, Fam109a, Fam110b, Fam123b, Fam126a, Fam126b, Fam131b, Fam13a, Fam13c, Fam155a, Fam160b1, Fam177a, Fam18b, Fam196a, Fam199x, Fam38b, Fam40a, Fam43a, Fam45a, Fam46a, Fam49a, Fam53b, Fam73a, Fam83f, Fap, Fbxl17, Fbxl20, Fbxo28, Fbxo32, Fbxo34, Fbxo42, Fbxo45, Fech, Fgd6, Fgf20, Fign, Fkbp14, Fkbp3, Fndc3a, Fndc3b, Fnip2, Fosl2, Foxa1, Foxb1, Foxd1, Foxg1, Foxo3, Foxp4, Frk, Frmd4a, Frmd6, Frmpd1, Frzb, Fst, Fubp1, Fxr1, Fyn, Fzd2, Fzd3, Gabrb1, Gadd45gip1, Galnt1, Galnt2, Galnt3, Galnt7, Galr1, Gas7, Gatm, Gclc, Gcnt2, Gda, Gdnf, Gfpt2, Gigyf1, Gigyf2, Gja1, Gjc2, Glcci1, Glce, Gldc, Gli2, Gm606, Gmeb1, Gmeb2, Gna13, Gnai2, Gnao1, Gnpda1, Golga1, Golga4, Got2, Gpd1l, Gpr124, Gpr125, Gpr157, Gpr30, Gpr45, Gpt2, Gramd2, Grb10, Grhl1, Grhl2, Gria2, Grm3, Grm5, Gtf2e2, Gtf2h1, Gtf3c4, Gxylt1, Gzf1, H2afy, Haus4, Hbs1l, Hdac9, Hecw1, Hephl1, Hic2, Hipk1, Hlf, Hnrnpab, Hnrnpul2, Hoxa1, Hoxa11, Hoxb3, Hoxb4, Hoxb8, Hpcal4, Hspa5, Htr4, Htra3, Ick, Ide, Idh1, Ier5, Iffo2, Ifnar2, Igf1, Igf1r, Igf2r, Igsf3, Ikzf2, Il1a, Ino80d, Insig2, Ip6k3, Ipmk, Irf4, Irs1, Irs4, Irx4, Isg20l2, Itfg1, Itga5, Itga8, Itga9, Itgb3, Itpk1, Itsn1, Ivns1abp, Jak1, Jakmip2, Jarid2, Jdp2, Josd1, Jph4, Kalrn, Katnal1, Kbtbd10, Kcna4, Kcnj12, Kcnmb2, Kcnn3, Kctd3, Kctd5, Kctd7, Kctd8, Kdm3a, Kdm6b, Khnyn, Kif21b, Kif3a, Klf10, Klf12, Klf13, Klf14, Klf8, Klf9, Klhdc2, Klhl20, Kpna3, Kpna6, Kras, Kremen1, Ksr1, Large, Larp1, Larp4, Lats2, Lclat1, Lcorl, Lepr, Lgi1, Lhx8, Lhx9, Lifr, Limch1, Lin28a, Lin28b, Lin7c, Lmbr1l, Lnp, Lonrf1, Lonrf3, Lox, Lpar3, Lpgat1, Lpp, Lrch2, Lrch3, Lrfn2, Lrp6, Lrrc17, Lrrc3, Lrrc40, Lrrc58, Lrrc8d, Lrrfip2, Lrrk2, Mab21l1, Maf, Mafg, Magi2, Magi3, Mal, Maml1, Man1a2, Man1b1, Map3k1, Map3k12, Map3k13, Map3k2, Map3k5, Mapk8, Mapkbp1, Mapre1, March4, March6, Marcks, Mast4, Mat2a, Mbd6, Mbnl1, Mbnl2, Mbnl3, Mboat1, Mbtps2, Mcf2l, Mdfic, Me1, Mecp2, Megf6, Meis2, Mex3b, Mex3c, Mfhas1, Mfsd11, Mfsd6, Mfsd7b, Mfsd7c, Mical1, Mid2, Mier2, Mier3, Mkl2, Mkrn3, Mll1, Mll2, Mll3, Mlxip, Mmd, Mnt, Mov10, Msi2, Mta1, Mtdh, Mthfd1l, Mttp, Mtx3, Mybl2, Myh11, Myo5a, Myo9b, Mzt1, Nacc2, Nadk, Nagpa, Nap1l3, Nap1l5, Nav1, Nav3, Ncald, Ncam1, Nck2, Ncor2, Ncs1, Ndel1, Necap1, Nedd4, Nedd4l, Nefl, Negr1, Neurl1b, Neurod1, Nf1, Nfat5, Nfatc3, Nfia, Nfib, Nhlh2, Nid1, Nipal1, Nipal4, Nkain2, Nkx2-2, Notch1, Nova1, Npy2r, Nr2f2, Nr3c1, Nr3c2, Nr5a2, Nr6a1, Nrbf2, Nrg3, Nrk, Nrp2, Nrxn3, Nsd1, Nsg1, Nt5e, Ntng1, Nuak1, Nufip2, Nus1, Ocln, Odz3, Ogfod1, Omg, Onecut2, Ovol2, Oxtr, P4ha1, P4ha2, Pafah1b2, Palm2, Pank3, Papola, Parp16, Pawr, Pax3, Pbrm1, Pcdh10, Pcdh17, Pcdh19, Pcdh20, Pcgf3, Pcgf5, Pcnxl2, Pdcd10, Pdcd5, Pdcl, Pde4d, Pde7a, Pdgfrb, Pdp2, Pdss1, Peli1, Peli2, Per2, Pfn2, Pggt1b, Pgm2, Phactr2, Phf13, Phf20, Phip, Phldb2, Phtf2, Pi4k2b, Picalm, Piga, Pik3cd, Pik3r2, Pip4k2a, Pip4k2b, Pitpnc1, Pitpnm2, Pitpnm3, Pknox2, Plag1, Plagl2, Plch1, Plcxd2, Plekhm3, Plekho2, Pls1, Plxna1, Plxna2, Plxnc1, Pnn, Pnpla2, Polr3e, Polr3g, Pon2, Pou4f2, Ppargc1a, Ppargc1b, Ppfia1, Ppfia2, Ppid, Ppp1r12a, Ppp1r14c, Ppp1r9a, Ppp2r1b, Ppp2r4, Ppp3ca, Ppp3cb, Ppp3r1, Pppde1, Pptc7, Prdm1, Prickle1, Prkaa2, Prlr, Prrg1, Prrt2, Prtg, Prune2, Psd3, Psmd7, Psme3, Ptgfrn, Ptp4a1, Ptpn13, Ptpn2, Ptpn21, Pus10, Pxn, Pydc4, Qk, R3hdm1, Rab15, Rab23, Rab27b, Rab32, Rab38, Rab8a, Rabgap1l, Rad23b, Rai14, Ranbp10, Ranbp9, Rap1b, Rap2c, Rapgef2, Rapgef4, Raph1, Rarb, Rarg, Rasa1, Rasa2, Rasal2, Rasd1, Rasgef1a, Rasgef1b, Rassf10, Rbfox1, Rc3h2, Rcc2, Reep1, Reep3, Rev1, Rev3l, Rffl, Rfx2, Rfx3, Rfx6, Rfx7, Rgs17, Rgs2, Rgs8, Rhebl1, Rimbp2, Rlf, Rlim, Rnf122, Rnf144b, Rnf157, Rnf165, Rnf2, Rnf220, Rnf24, Rnf44, Rnmt, Rock2, Rod1, Rprd2, Rps6ka2, Rps6ka5, Rqcd1, Rrad, Rtn4r, Runx1, Runx2, Rwdd3, Rwdd4a, Sall4, Samd4, Samd8, Satb1, Sbf1, Sbk1, Scamp1, Scara5, Scn1a, Scn2a1, Scn3a, Scn8a, Scn9a, Scyl3, Sdad1, Sec14l2, Sec23a, Sec24a, Sec61a2, Sec62, Sel1l3, Sema3a, Sema6b, Sema6d, Sept6, Sept7, Sept8, Serpine1, Setd5, Setd7, Sgcb, Sgk3, Sgms2, Sh2b3, Sh3pxd2a, Sh3rf1, Shoc2, Siah2, Sik3, Sirt1, Six1, Six4, Skil, Skp2, Slc12a6, Slc1a2, Slc22a23, Slc30a4, Slc35a3, Slc35a5, Slc35c1, Slc35d3, Slc35f1, Slc35f4, Slc36a1, Slc38a1, Slc38a2, Slc38a4, Slc38a7, Slc41a2, Slc4a7, Slc5a11, Slc5a3, Slc6a6, Slc6a9, Slc7a10, Slc7a11, Slc7a6, Slc9a8, Slco5a1, Smad1, Smap1, Smarcd2, Snai1, Snai2, Snx16, Snx18, Snx30, Snx33, Sobp, Socs1, Socs3, Socs6, Sos1, Sox11, Sox12, Sox9, Sp4, Spast, Spcs3, Spen, Spty2d1, Srgap3, Srsf7, Ssbp2, Ssx2ip, St8sia4, Stac, Stag2, Stc1, Stim2, Stk35, Stk38l, Stk39, Stox2, Strbp, Stx16, Stx2, Stxbp5, Styx, Surf4, Syn2, Syngr3, Sypl, Tab3, Tada2b, Taf4b, Taok1, Tasp1, Tbc1d10b, Tbc1d15, Tbc1d2b, Tbl1xr1, Tbpl1, Tbx22, Tcf7, Tcp11l1, Tcp11l2, Tdg, Tet1, Tet3, Thbs2, Tia1, Timp2, Timp3, Tm9sf3, Tmcc1, Tmed10, Tmed2, Tmeff1, Tmem121, Tmem170b, Tmem181a, Tmem229a, Tmem87a, Tmod2, Tmod3, Tnik, Tnrc6a, Tnrc6b, Tnrc6c, Tnxb, Tor1b, Tpm4, Traf6, Trim33, Trim36, Trim9, Trio, Trip12, Tro, Trpm7, Trps1, Tsga14, Tspan2, Tspan33, Tspyl4, Ttbk1, Ttc39a, Ttll7, Tubgcp3, Tulp4, Tusc3, Twf1, Ube2d2, Ube2d3, Ube2g1, Ube2i, Ube2j1, Ube2o, Ube2ql1, Ube2r2, Ube2v2, Ube3c, Ubn1, Ubn2, Ugt8a, Unc5c, Unc5d, Unkl, Usp14, Usp2, Slc35f1, Slc35f4, Slc36a1, Slc38a1, Slc38a2, Slc38a4, Slc38a7, Slc41a2, Slc4a7, Slc5a11, Slc5a3, Slc6a6, Slc6a9, Slc7a10, Slc7a11, Slc7a6, Slc9a8, Slco5a1, Smad1, Smap1, Smarcd2, Snai1, Snai2, Snx16, Snx18, Snx30, Snx33, Sobp, Socs1, Socs3, Socs6, Sos1, Sox11, Sox12, Sox9, Sp4, Spast, Spcs3, Spen, Spty2d1, Srgap3, Srsf7, Ssbp2, Ssx2ip, St8sia4, Stac, Stag2, Stc1, Stim2, Stk35, Stk38l, Stk39, Stox2, Strbp, Stx16, Stx2, Stxbp5, Styx, Surf4, Syn2, Syngr3, Sypl, Tab3, Tada2b, Taf4b, Taok1, Tasp1, Tbc1d10b, Tbc1d15, Tbc1d2b, Tbl1xr1, Tbpl1, Tbx22, Tcf7, Tcp11l1, Tcp11l2, Tdg, Tet1, Tet3, Thbs2, Tia1, Timp2, Timp3, Tm9sf3, Tmcc1, Tmed10, Tmed2, Tmeff1, Tmem121, Tmem170b, Tmem181a, Tmem229a, Tmem87a, Tmod2, Tmod3, Tnik, Tnrc6a, Tnrc6b, Tnrc6c, Tnxb, Tor1b, Tpm4, Traf6, Trim33, Trim36, Trim9, Trio, Trip12, Tro, Trpm7, Trps1, Tsga14, Tspan2, Tspan33, Tspyl4, Ttbk1, Ttc39a, Ttll7, Tubgcp3, Tulp4, Tusc3, Twf1, Ube2d2, Ube2d3, Ube2g1, Ube2i, Ube2j1, Ube2o, Ube2ql1, Ube2r2, Ube2v2, Ube3c, Ubn1, Ubn2, Ugt8a, Unc5c, Unc5d, Unkl, Usp14, Usp2, Usp37, Usp45, Usp47, Usp48, Usp49, Vamp3, Vapa, Vat1, Vat1l, Vav3, Vip, Vkorc1l1, Vopp1, Vps26b, Vps33a, Wdr26, Wdr44, Wdr7, Wdr82, Wipf1, Wipf3, Wnt7b, Wwtr1, Xkr4, Xpo1, Xpr1, Yaf2, Ybx1, Yod1, Ypel2, Ypel5, Ythdc1, Ythdf3, Ywhag, Ywhaz, Zbtb10, Zbtb11, Zbtb34, Zbtb39, Zbtb41, Zbtb44, Zbtb7a, Zcchc14, Zcchc2, Zcchc24, Zdhhc17, Zdhhc20, Zdhhc21, Zeb2, Zfc3h1, Zfp148, Zfp238, Zfp275, Zfp280b, Zfp36l2, Zfp507, Zfp518a, Zfp521, Zfp608, Zfp644, Zfp704, Zfp706, Zfp711, Zfp746, Zfp770, Zfyve26, Zic4, Zmynd8, Znrf1, Zswim6 |
| **mir-344c-3p:** |
| Ahcyl2, Appl1, Arih2, Cbx4, Cbx5, Ccdc142, Creb1, Dsc1, Eln, Epb4.1l1, Esrrg, Fbxo11, Gm106, Gpd2, Ino80d, Kcne4, Lrrc8a, Med19, Nrn1, Nufip2, Olfml2b, Onecut2, Peg3, Pou4f1, Rad23b, Satb1, Scrt1, Tcf23, Wasf2, Zzz3 |
| **mir-383-5p:** |
| 4632428N05Rik, 4933403F05Rik, Acvr1b, Ap3m1, Arid2, Brcc3, Brip1, Clec16a, Cnih, Col4a3, Cry2, Ctnnal1, Dnmt3a, Dpysl2, Dtx4, Eif4g2, Elavl2, Epas1, Epn2, Fat3, Fos, Fryl, Gadd45g, Gfer, Gldn, Gm1337, Hnrnpa1, Il1rap, Immt, Irf1, Kif5c, Kirrel3, Mal2, Mettl7a1, Mga, Mmp24, Mtap7d1, Nfat5, Nnat, Nup153, Pank1, Pcdh12, Pcdh17, Pgm2l1, Phka1, Pldn, Prdx3, Ptpre, Qk, Rapgef6, Rmnd5a, Rsf1, Runx1, Samd4b, Samd8, Shmt2, Sla, Slc1a2, Slc35a3, Sox11, Sp7, Sppl3, Spred3, Stk38l, Taf9b, Taok3, Tbc1d5, Trim33, Ube2r2, Wipf2, Zfp647, Zfp706 |
| **mir-433-3p:** |
| 2610101N10Rik, 3830406C13Rik, Aak1, Acad8, Actr3, Adpgk, Agps, Ankrd12, Ankrd52, Ano5, Arl13b, Arrdc4, Azin1, B4galt3, Bard1, C030046E11Rik, Ccar1, Ccdc43, Ccdc47, Cdc27, Cdk5r1, Cenpj, Cep135, Chd9, Chl1, Clint1, Cltc, Cox6b1, Cox8a, Creb1, Crlf3, Cul5, Cycs, D15Ertd621e, Ddx19a, Dnajc5, E2f3, Edn3, Elovl2, Eny2, Epc2, Epha7, Epn2, Foxo6, Foxp2, Gabpa, Galnt1, Gata3, Grb2, Hdlbp, Hivep1, Hnrnpa1, Hnrnpr, Hoxa1, Hoxa5, Hoxc8, Hs3st3b1, Ints2, Kcnd3, Kcnv1, Kras, Ky, Lamc1, Lrrc58, Lsm11, Mark3, Mbnl1, Med13, Mier1, Mll1, Mmd, Mn1, Mxi1, Myh9, Nab1, Npy1r, Nr2f6, Nr3c1, Nsd1, Nsl1, Olfm3, Paip1, Pak4, Pcbp2, Pccb, Ppp3r1, Prdm8, Qk, Rfx7, Rhobtb2, Rhoq, Rnf24, Rraga, Rsbn1, Scaf1, Scn1b, Scn3b, Scoc, Sema6a, Serbp1, Sft2d2, Sgms2, Slc16a1, Slc17a5, Smg5, Sntg1, Snx11, Sorbs1, Sparc, Srp54c, Ssbp2, Stam, Stox2, Strbp, Stxbp3a, Styx, Syncrip, Tbc1d19, Tia1, Tmed5, Tmem33, Tmem50a, Tnrc6b, Tnrc6c, Top1, Triap1, Trip12, Ttc13, Ubr1, Usp14, Ust, Wac, Wdr45l, Ypel5, Ythdf3, Ywhag, Ywhaz, Zcchc6, Zdhhc7, Zfhx4, Zfp300, Zzz3 |
| **mir-451a:** |
| Cab39, Cox10, Fign, Gyk, Samd4b, Tctex1d4, Trim66, Ythdf2 |
| **mir-455-5p:** |
| Gjd2, Itga1, Ktelc1, Nup153, Ppp3r2, Rbm14, Slit2, Ssr1, Tox3, Zfp518 |
| **mir-541-5p:** |
| 9930021J03Rik, Acvr1b, Adcy6, Apbb3, Aqp4, Arid2, Atxn7, Braf, Cbx5, Dcc, Diap2, Dnajb1, Dnajb12, Eif3j, Esr1, Faf2, Fbxw7, Gab1, Glyctk, Gnai3, Hmga2, Hoxd10, Ids, Kcnj10, Kcnmb2, Khdrbs3, Kpna1, Lrrc19, Mat2a, Mbnl1, Napb, Ndufb8, Pard3, Pcdh19, Pgm3, Rnf38, Rorb, Runx1t1, Slc5a3, Slc6a6, Spag9, St13, Stk3, Tbc1d9b, Tcf4, Tgfbr3, Thtpa, Trps1, Ttc14, Wbp7, Zfx, Zkscan1 |
| **mir-678:** |
| Mpz, Gpr3 |
| **mir-690:** |
| 1110004F10Rik, 1600021P15Rik, 1700037H04Rik, 2310022B05Rik, 2510009E07Rik, 2810046L04Rik, 9930021J03Rik, Abcf3, Abi2, Ablim3, Acsl1, Adam23, Agpat9, Agps, AI597479, Akap6, Akt2, Amfr, Ankrd13a, Arf6, Arhgdia, Arhgef7, Atad1, Atg3, Atmin, Atp11a, Atxn1l, Axin2, B3galt1, Bbx, BC017647, BC048403, Bicd1, Bmpr1a, Brsk2, Brwd3, Bsn, Btaf1, C1ql3, Cadm2, Cbln2, Cbx5, Ccdc117, Cd276, Cdc27, Cdc6, Cdon, Chd6, Clic5, Cmtm6, Cnn3, Cnot6, Cnrip1, Cog3, Cpeb3, Cpsf6, Creb1, Csnk1g1, Ctnnb1, Cyp1b1, Dcc, Dclk1, Dcun1d4, Ddost, Dkc1, Drd1a, E2f7, Efnb2, Egr1, Eif3j, Eif4g2, Elovl2, Enah, Entpd7, Epb4.1l3, Erf, Etv3, Fbxl7, Fbxw2, Fscn1, Gabbr2, Gabrb2, Gcn1l1, Gdi2, Gdpd1, Gemin5, Glrx5, Gm608, Gna13, Golt1b, Gpr22, Gpr85, Grm5, Hcfc1, Hiat1, Hint3, Hook3, Huwe1, Igf2r, Ikzf2, Impa1, Ing3, Ippk, Jag1, Jarid2, Kcnh1, Kif21b, Kif5a, Kras, Ktn1, Lamc1, Mapk4, Mbnl1, Mbnl3, Mccc2, Med26, Megf11, Meis1, Mfap3l, Mgat4a, Mier1, Mll1, Mllt3, Mn1, Mov10, Mtdh, Mtpn, Mylip, Myo9b, Nab1, Nap1l4, Ncor2, Nedd4l, Nefm, Nfatc1, Nfia, Nfib, Nrd1, Nrp1, Nudt11, Obfc2a, Odz4, Pa2g4, Padi3, Pafah1b2, Pag1, Pak2, Pak3, Pcgf3, Peg3, Pid1, Pitpnm2, Plagl2, Plk3, Plod2, Ppl, Ppm1g, Ppp1r9a, Prkca, Prkcz, Prrg3, Pten, Ptgfrn, Ptp4a1, Ptp4a2, Qrfp, Rab11a, Rab11b, Rab3c, Rab5b, Rad54l2, Ranbp3, Rap1b, Rap2c, Rapgef6, Raver1, Rnf144a, Rnf24, Rps27l, Rras2, Sbno1, Scn7a, Sdcbp, Setd7, Sgtb, Shroom3, Slc12a2, Slc16a1, Slc18a2, Slc35a1, Slc41a2, Slk, Snrk, Socs2, Sox9, Spg20, Spred2, Ssh3, Stam2, Stat3, Stom, Suclg2, Suv420h1, Syncrip, Tbc1d14, Tbc1d15, Tbc1d8b, Tceb3, Tial1, Tiprl, Tmem189, Tmem87b, Tpcn1, Tpd52l2, Trove2, Tspan14, Ttpal, Txlna, Ube2e3, Ube2o, Ubl3, Uhrf1bp1, Ulk2, Unc5b, Vrk1, Wac, Wasf1, Wdfy1, Wdr26, Wdr82, Wdtc1, Ythdf1, Zbed4, Zbtb5, Zdhhc15, Zdhhc8, Zeb1, Zeb2, Zfand1, Zfand3, Zfp287, Zfp362, Zfp697, Zmiz1, Zxdb |
| **miR-883b-5p:** |
| Abat, Abca5, Afap1, Asxl2, Bag5, Bap1, BC013712, Btg2, Bzw1, Camk1d, Camk2a, Camk2n1, Camsap1l1, Cask, Ccdc88a, Cdh20, Clcn4-2, Cplx2, Cpne6, Crebl2, Ctbp2, Ctnnal1, D17Wsu92e, D1Pas1, Dclk1, Ddx3x, Dnajc1, Dpy19l1, Dusp7, E130309F12Rik, Eif4g2, Esrrg, Ets1, Fmnl2, Frk, Frmpd4, Galnt7, Gcnt2, Ggta1, Git2, Glipr2, Gpr173, Grm5, Hipk3, Hlf, Hmbox1, Hoxb5, Kcnj12, Kif3c, Leng8, Lnp, Map3k2, Mark3, Mboat2, Mbtps2, Med1, Med13, Mga, Mtf1, Mycbp, Myo18a, Nab1, Ndst1, Nup50, Olfm3, Pak6, Pcdh9, Pds5b, Phactr1, Phc1, Pik3cg, Pip4k2b, Pkd1, Prickle2, Prkar2a, Psme3, Qk, Rab35, Rap2c, Rdbp, Rere, Rprd1a, Rs1, Rtn3, Rybp, Sec24c, Sel1l, Sertad2, Sidt2, Slc30a7, Slitrk4, Smg7, Sox11, Spg20, Srf, Stag1, Stk35, Strbp, Syncrip, Syt1, Taf4a, Tcf7l2, Thsd7b, Tiparp, Trip4, Ttc9, Txlna, Ube2d1, Ube2r2, Uqcc, Usp19, Vamp5, Vat1, Vwa3a, Zfp689, Zfp706 |

**Supplementary Table 2.** List of gene targets for the significant and annotated miRNAs used in IPA analysis

|  |  |  | | **Rel. Luc. Activity 1** | | **Rel. Luc. Activity 2** | | | **mir-30e-mediated exogenous effect on pEZX-MT06/EFNA3-3'UTR or**  **pEZX-MT06**  **A=(1)/(2) / B=(3)/(4)** | | | **mir-30e-mediated exogenous effect on EFNA3-3'UTR**  **= A/B** | | | **% change in gene expression** | | |
| --- | --- | --- | --- | --- | --- | --- | --- | --- | --- | --- | --- | --- | --- | --- | --- | --- | --- |
| **HEK cells** | 1 | (1) pEZX-MT06/EFNA3-3'UTR + mir-30e | | 0,030 | | 0,030 | | | A= 0,96 | | |  | | |  | | |
|  |  | (2) pEZX-MT06/EFNA3-3'UTR + neg.miRNA | | | 0,031 | | | 0,031 | | |  | | | **0,85** | | | 15% decrease |
|  |  | (3) pEZX-MT06 + mir-30e | 0,076 | | | | 0,076 | | | B= 1,13 | | |  | | |  | |
|  |  | (4) pEZX-MT06 + neg.miRNA | 0,067 | | | | 0,066 | | |  | | |  | | |  | |
|  |  |  |  |  |  |  |  |  |  |  |  |  |  |  |  |  |  |
|  | 2 | (1) pEZX-MT06/EFNA3-3'UTR + mir-30e | 0,027 | | | | 0,027 | | | A= 0,89 | | |  | | |  | |
|  |  | (2) pEZX-MT06/EFNA3-3'UTR + neg.miRNA | 0,030 | | | | 0,030 | | |  | | | **0,89** | | | 11% decrease | |
|  |  | (3) pEZX-MT06 + mir-30e | 0,059 | | | | 0,059 | | | B= 1,00 | | |  | | |  | |
|  |  | (4) pEZX-MT06 + neg.miRNA | 0,059 | | | | 0,059 | | |  | | |  | | |  | |
|  |  |  |  |  |  |  |  |  |  |  |  |  |  |  |  |  |  |
|  | 3 | (1) pEZX-MT06/EFNA3-3'UTR + mir-30e | 0,024 | | | | 0,024 | | | A= 0,86 | | |  | | |  | |
|  |  | (2) pEZX-MT06/EFNA3-3'UTR + neg.miRNA | 0,028 | | | | 0,028 | | |  | | | **0,88** | | | 12% decrease | |
|  |  | (3) pEZX-MT06 + mir-30e | 0,053 | | | | 0,052 | | | B= 0,98 | | |  | | |  | |
|  |  | (4) pEZX-MT06 + neg.miRNA | 0,053 | | | | 0,054 | | |  | | |  | | |  | |
|  |  |  | |  | |  | | |  | | |  | | |  | | |
| **HeLa cells** | 4 | (1) pEZX-MT06/EFNA3-3'UTR + mir-30e | | 0,022 | | 0,021 | | | A= 1,09 | | |  | | |  | | |
|  |  | (2) pEZX-MT06/EFNA3-3'UTR + neg.miRNA | | | 0,020 | | | 0,020 | | |  | | | **0,86** | | | 14% decrease |
|  |  | (3) pEZX-MT06 + mir-30e | 0,051 | | | | 0,050 | | | B= 1,26 | | |  | | |  | |
|  |  | (4) pEZX-MT06 + neg.miRNA | 0,040 | | | | 0,040 | | |  | | |  | | |  | |
|  |  |  |  |  |  |  |  |  |  |  |  |  |  |  |  |  |  |
|  | 5 | (1) pEZX-MT06/EFNA3-3'UTR + mir-30e | 0,017 | | | | 0,017 | | | A= 1,17 | | |  | | |  | |
|  |  | (2) pEZX-MT06/EFNA3-3'UTR + neg.miRNA | 0,015 | | | | 0,015 | | |  | | | **0,86** | | | 14% decrease | |
|  |  | (3) pEZX-MT06 + mir-30e | 0,040 | | | | 0,041 | | | B= 1,37 | | |  | | |  | |
|  |  | (4) pEZX-MT06 + neg.miRNA | 0,030 | | | | 0,029 | | |  | | |  | | |  | |
|  |  |  |  |  |  |  |  |  |  |  |  |  |  |  |  |  |  |
|  | 6 | (1) pEZX-MT06/EFNA3-3'UTR + mir-30e | 0,013 | | | | 0,013 | | | A= 1,14 | | |  | | |  | |
|  |  | (2) pEZX-MT06/EFNA3-3'UTR + neg.miRNA | 0,012 | | | | 0,012 | | |  | | | **0,92** | | | 8% decrease | |
|  |  | (3) pEZX-MT06 + mir-30e | 0,029 | | | | 0,030 | | | B= 1,23 | | |  | | |  | |
|  |  | (4) pEZX-MT06 + neg.miRNA | 0,024 | | | | 0,024 | | |  | | |  | | |  | |

**Supplementary Table 3.** Raw data for the luciferase reporter gene assay. Luciferase assay used mir-30e-5p and EFNA3 3’UTR. Mir-30e-5p leads to a downregulation of EFNA3 as illustrated by an average 12% reduction in luciferase activity (normalized). The normalization procedure was based on Campos-Melo et al. 2014^8^ ensuring that confounding factors classically arising in luciferase assays are controlled for. We first normalized the Firefly luciferase activity with the Renilla luciferase activity (transfection control / not shown). The computed “Relative luciferase activity” is presented in this table (2 biological replicated per condition). We then normalized the Relative luciferase activity against a) the cellular effects (e.g., RNA binding proteins, endogenous miRNAs, etc.) on luciferase activity and b) against the exogenous effects of mir-30e-5p on the luciferase coding sequences. See Supplementary Methods for further details.

| **Significantly downregulated miRNAs** | | | |
| --- | --- | --- | --- |
| **Accession Number** | **Transcript ID** | ***p*-value** | **Fold-Change (HFD vs. CD)** |
| MIMAT0001632 | mmu-miR-451a | 0.00716281 | -1.73801 |
| MIMAT0017281 | mmu-miR-511-3p | 0.0232533 | -1.67302 |
| MIMAT0014928 | mmu-miR-344c-3p | 0.0336578 | -1.58691 |
| MIMAT0017209 | mmu-miR-541-3p | 0.0216328 | -1.53285 |
| MIMAT0003469 | mmu-miR-690 | 0.00618111 | -1.52981 |
| MIMAT0017040 | mmu-miR-350-5p | 0.0245193 | -1.34234 |
| MIMAT0014864 | mmu-miR-3078-5p | 0.0416764 | -1.33395 |
| MIMAT0003485 | mmu-miR-455-5p | 0.00250818 | -1.31711 |
| MIMAT0004850 | mmu-miR-883b-5p | 0.0340633 | -1.31545 |
| MIMAT0017210 | mmu-miR-547-5p | 0.0313436 | -1.26247 |
| MIMAT0000648 | mmu-miR-10a-5p | 0.0437868 | -1.25814 |
| MIMAT0016988 | mmu-miR-144-5p | 0.0121198 | -1.25327 |
| MIMAT0000248 | mmu-miR-30e-5p | 0.0303815 | -1.20456 |
| **Significantly upregulated miRNAs** | | | |
| **Accession Number** | **Transcript ID** | ***p*-value** | **Fold-Change (HFD vs. CD)** |
| MIMAT0020627 | mmu-miR-5119 | 0.0296401 | 1.21053 |
| MIMAT0003170 | mmu-miR-541-5p | 0.0324367 | 1.21786 |
| MIMAT0000748 | mmu-miR-383-5p | 0.0117387 | 1.22856 |
| MIMAT0004628 | mmu-miR-21a-3p | 0.0281818 | 1.25431 |
| MIMAT0001420 | mmu-miR-433-3p | 0.0145485 | 1.27067 |
| MIMAT0004826 | mmu-miR-146b-3p | 0.0128916 | 1.27534 |
| MIMAT0003452 | mmu-miR-678 | 0.0369783 | 1.32879 |
| MIMAT0017275 | mmu-miR-467c-3p | 0.0260913 | 1.41821 |
| MIMAT0017052 | mmu-miR-210-5p | 0.0311217 | 1.51463 |

**Supplementary Table 4.** List of miRNAs used for DIANA analysis.

| **List of miRNAs affected by HFD and with target-genes in the Axon guidance pathway** | | | |
| --- | --- | --- | --- |
| *Total: 15 miRNAs and 51 different genes; p-value for the Axon guidance pathway: p=1.29 10^25* | | | |
|  | | | |
| **MiRNA** | **Number of target-genes** | ***p*-value for miRNA** | **Fold-Change for miRNA (HFD vs. CD)** |
| miR-10a-5p | 2 | 0.0437868 | -1.25814 |
| miR-210-5p | 1 | 0.0311217 | 1.51463 |
| miR-21-3p | 4 | 0.0281818 | 1.25431 |
| miR-3078-5p | 2 | 0.0416764 | -1.33395 |
| miR-30e-5p | 21 | 0.0303815 | -1.20456 |
| miR-344c-3p | 1 | 0.0336578 | -1.58691 |
| miR-350-5p | 11 | 0.0245193 | -1.34234 |
| miR-383-5p | 1 | 0.0117387 | 1.22856 |
| miR-433-3p | 4 | 0.0145485 | 1.27067 |
| miR-511-3p | 6 | 0.0232533 | -1.67302 |
| miR-5119 | 1 | 0.0296401 | 1.21053 |
| miR-547-5p | 1 | 0.0313436 | -1.26247 |
| miR-690 | 2 | 0.00618111 | -1.52981 |
| miR-883b-5p | 2 | 0.0340633 | -1.31545 |
| miR-467c-3p | 12 | 0.0260913 | 1.41821 |

**Supplementary Table 5.** List of miRNAs affected by HFD and with target-genes in the Axon guidance pathway. The table gives, for each miRNA affected by HFD, the number of target genes within the axon guidance pathway, as well as the p-value and fold change for this miRNA as an effect of HFD vs. CD treatment.

.


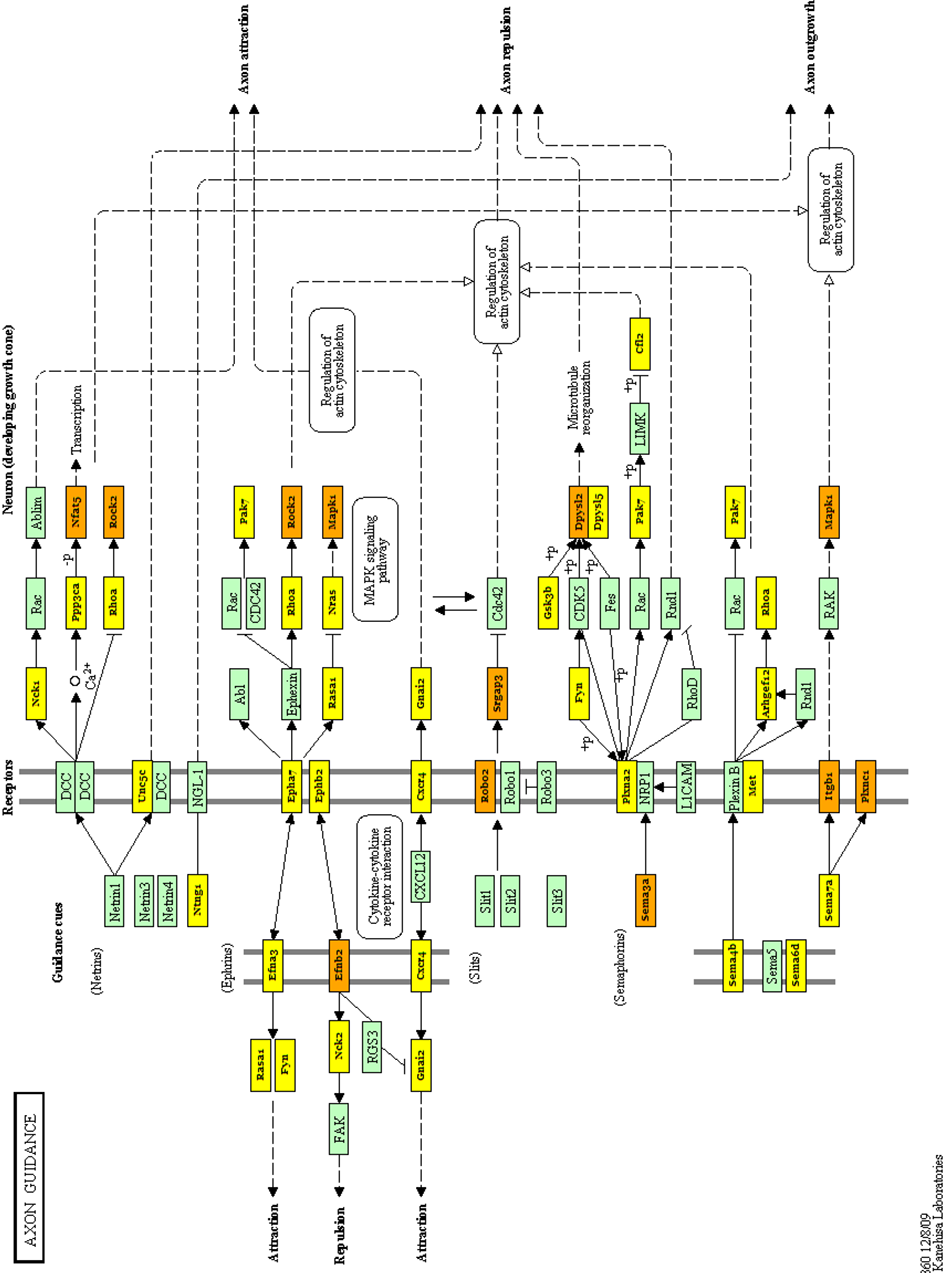


**Supplementary Figure 1**. The server provides in the KEGG^9,10^ pathway ‘axon guidance’ three levels of gene labeling: yellow (gene targeted by 1 selected miRNA), orange (gene targeted by >1 selected miRNAs), green (gene not targeted by selected miRNAs)

**References**

1 Spear, L. P. The adolescent brain and age-related behavioral manifestations. *Neurosci Biobehav Rev* **24**, 417-463 (2000).

2 Boitard, C. *et al.* Juvenile, but not adult exposure to high-fat diet impairs relational memory and hippocampal neurogenesis in mice. *Hippocampus* **22**, 2095-2100, doi:10.1002/hipo.22032 (2012).

3 Labouesse, M. A. *et al.* Hypervulnerability of the adolescent prefrontal cortex to nutritional stress via reelin deficiency. *Mol Psychiatry* **22**, 961-971, doi:10.1038/mp.2016.193 (2017).

4 Labouesse, M. A., Stadlbauer, U., Langhans, W. & Meyer, U. Chronic high fat diet consumption impairs sensorimotor gating in mice. *Psychoneuroendocrinology* **38**, 2562-2574, doi:10.1016/j.psyneuen.2013.06.003 (2013).

5 Rakic, P., Bourgeois, J. P. & Goldman-Rakic, P. S. Synaptic development of the cerebral cortex: implications for learning, memory, and mental illness. *Prog Brain Res* **102**, 227-243, doi:10.1016/S0079-6123(08)60543-9 (1994).

6 Tau, G. Z. & Peterson, B. S. Normal development of brain circuits. *Neuropsychopharmacology* **35**, 147-168, doi:10.1038/npp.2009.115 (2010).

7 Baddeley, A. Working memory: looking back and looking forward. *Nat Rev Neurosci* **4**, 829-839, doi:10.1038/nrn1201 (2003).

8 Campos-Melo, D., Droppelmann, C. A., Volkening, K. & Strong, M. J. Comprehensive luciferase-based reporter gene assay reveals previously masked up-regulatory effects of miRNAs. *Int J Mol Sci* **15**, 15592-15602, doi:10.3390/ijms150915592 (2014).

9 Kanehisa, M., Furumichi, M., Tanabe, M., Sato, Y. & Morishima, K. KEGG: new perspectives on genomes, pathways, diseases and drugs. *Nucleic Acids Res* **45**, D353-D361, doi:10.1093/nar/gkw1092 (2017).

10 Kanehisa, M., Sato, Y., Kawashima, M., Furumichi, M. & Tanabe, M. KEGG as a reference resource for gene and protein annotation. *Nucleic Acids Res* **44**, D457-462, doi:10.1093/nar/gkv1070 (2016).
